# Lithospheric flexure and rheology determined by climate cycle markers in the Corinth Rift

**DOI:** 10.1038/s41598-018-36377-1

**Published:** 2019-03-07

**Authors:** Gino de Gelder, David Fernández-Blanco, Daniel Melnick, Guillaume Duclaux, Rebecca E. Bell, Julius Jara-Muñoz, Rolando Armijo, Robin Lacassin

**Affiliations:** 1 Institut de Physique du Globe de Paris, Sorbonne Paris Cité, Université Paris Diderot, UMR 7154 CNRS, 1 Rue Jussieu, F-75005 Paris, France; 2 Institut für Erd- und Umweltwissenschaften, Universität Potsdam, Karl-Liebknecht-Strasse 24, 14476 Potsdam, Germany; 3 Instituto de Ciencias de la Tierra, Universidad Austral de Chile, Casilla 567, Valdivia, Chile; 4 Université Côte d’Azur, UNS, CNRS, OCA, IRD, Géoazur, 06560 Valbonne France; 5 Basins Research Group (BRG), Department of Earth Science & Engineering, Imperial College, Prince Consort Road, London, SW7 2BP UK

## Abstract

Geomorphic strain markers accumulating the effects of many earthquake cycles help to constrain the mechanical behaviour of continental rift systems as well as the related seismic hazards. In the Corinth Rift (Greece), the unique record of onshore and offshore markers of Pleistocene ~100-ka climate cycles provides an outstanding possibility to constrain rift mechanics over a range of timescales. Here we use high-resolution topography to analyse the 3D geometry of a sequence of Pleistocene emerged marine terraces associated with flexural rift-flank uplift. We integrate this onshore dataset with offshore seismic data to provide a synoptic view of the flexural deformation across the rift. This allows us to derive an average slip rate of 4.5–9.0 mm·yr^−1^ on the master fault over the past ~610 ka and an uplift/subsidence ratio of 1:1.1–2.4. We reproduce the observed flexure patterns, using 3 and 5-layered lithospheric scale finite element models. Modelling results imply that the observed elastic flexure is produced by coseismic slip along 40–60° planar normal faults in the elastic upper crust, followed by postseismic viscous relaxation occurring within the basal lower crust or upper mantle. We suggest that such a mechanism may typify rapid localised extension of continental lithosphere.

## Introduction

**Figure 1:**
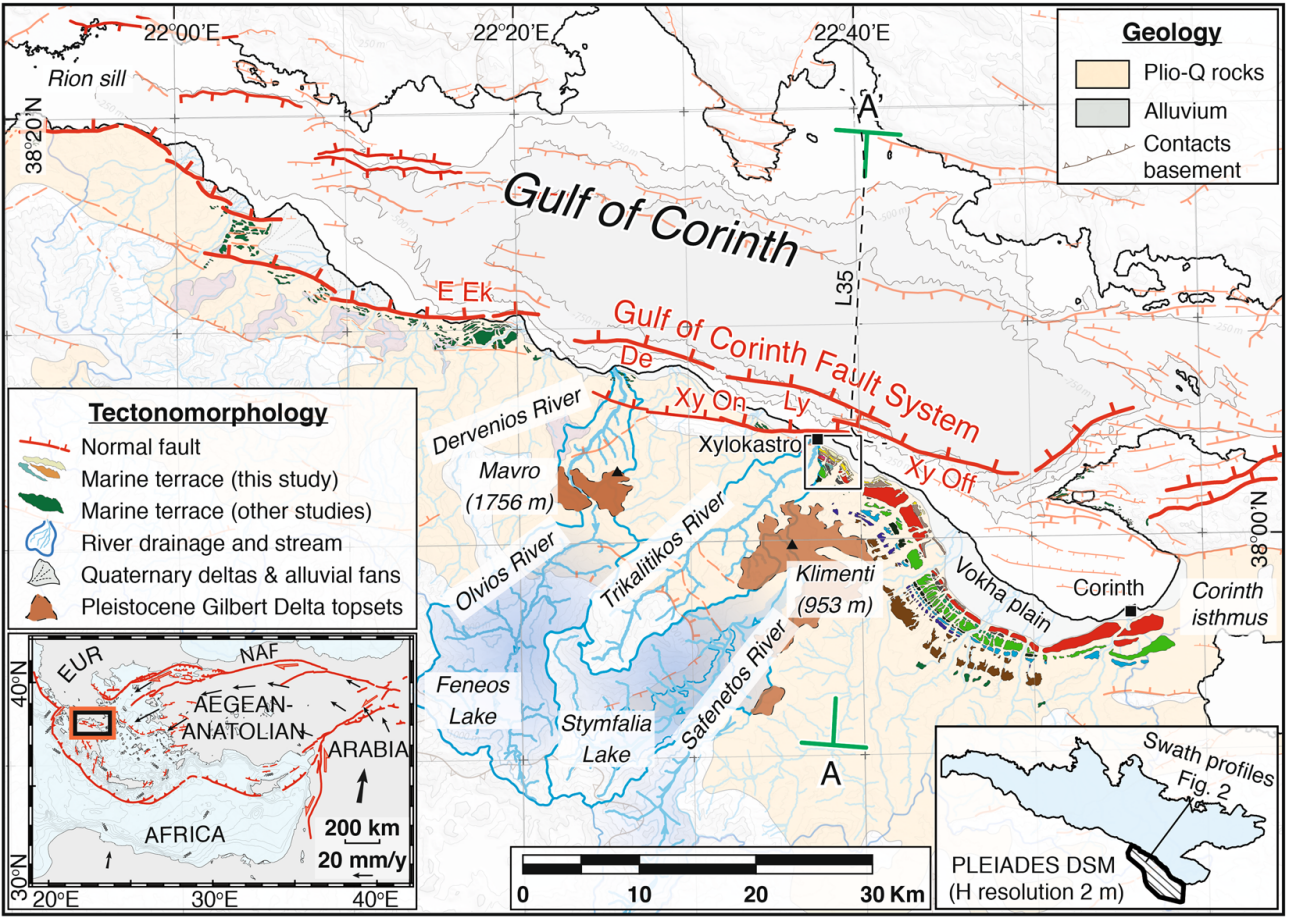
Active tectonics in the Gulf of Corinth. Solid box outlines location of Fig. 2, and A-A’ indicates cross-section location for Fig. 3. Faults mentioned in the text are the East Eliki (E Ek), Dervenios (De), Lykoporia (Ly) and Xylokastro on- and offshore faults (Xy On and Xy Off). Marine terraces from other studies have been adopted from refs^10,13,71^. Map was made using MAPublisher version 9.8 (http://www.avenza.com/help/mapublisher/9.8/).

Extension in continental rifts is characterised by normal faulting in the seismogenic upper crust, and a combination of brittle and/or ductile deformation in the underlying lower crust and upper mantle^1^. Our primary understanding of lithospheric extension mechanisms and rheological layering within such rifts is based on observations of the earthquake cycle at short timescales (10°–10^3^ yr)^2–5^, or of evolved mature rift systems formed over geological timescales (10^6^–10^8^ yr)^6–9^. However, observations of deformation in modern active continental rifts, as documented by geology and geomorphology thus integrating many earthquake cycles, allows for incorporating crustal deformation at spatial scales of tens of km and on timescales of 10^4^–10^6^ yr. Here we aim to characterize lithospheric rheology and extension mechanisms at the young and very fast-evolving Corinth Rift in Greece (Fig. 1), a currently asymmetric rift born in the Plio-Pleistocene^10,11^. Along the southern rift shoulder, a 130-km-long north-dipping active major fault system, composed of en-echelon fault segments with lengths of ~10–20 km (Fig. 1), controls the rift present-day morphology. Slip on these faults, and possibly on currently inactive ones, have resulted in upward flexure associated with >1.75-km of footwall uplift, and downwarped flexure associated with >3-km of hanging-wall subsidence, as evidenced from the uplifted Mavro delta^10^ and offshore basement depth^12^, respectively. Onshore, footwall flexural uplift has deformed a sequence of emerged Pleistocene marine terraces correlated with 100-ka glacio-eustatic climate cycles and dramatically modified the fluvial drainage network. Offshore, bathymetric sills^13,14^ controlled sedimentation in the Gulf as a function of the same glacio-eustatic cycles, switching rhythmically from lacustrine environment during sea-level lowstands to marine during highstands^15,16^.

We take advantage of the Corinth Rift’s exceptional geological setting and combined geomorphic/stratigraphic record, and analyse at high resolution the uplifted marine terraces between the towns of Corinth and Xylokastro (Fig. 1). In this site, previous studies analysed topographic maps and individual profiles and revealed the large-scale deformation of the terraces marked by a systematic elevation decrease with distance from the main north-dipping fault system^10,17,18^. Here we improve this onshore record of the deformation and link it directly to the now well-resolved tectono-stratigraphic framework deduced from offshore studies in the gulf (Nixon *et al*.^19^ and references therein). We use a high-resolution Digital Surface Model (DSM) to resolve more accurately the terrace uplift and onshore flexural pattern, and complement this analysis with depth-converted offshore seismic data^20^. This exceptional dataset provides a unique integrated view of the flexure resulting from continental rifting. Our onshore-offshore description of the flexural uplift and subsidence is best analysed using a new modelling approach at crustal-scale that refines previous attempts that were able to reproduce the uplift pattern of the terraces using an extremely weak (E = 0.1 GPa) elastic crust^10^ or ignoring the elastic stress field and normal faulting in the upper crust^21^. We use an updated numerical modelling approach that allows us to resolve the primary rheological parameters controlling lithospheric deformation in the young rift system. This study provides new constraints on the dynamics of the Corinth Rift system, critical for understanding both active deformation during early continental rifting and its controlling mechanisms at thousand- to million-years timescales. We propose that those same mechanisms may be responsible for the observed elastic flexure in active normal faults and young rift systems worldwide.

## Uplifted Marine Terraces

The outstanding flight of uplifted marine terraces in the Corinth Rift^17,18^ has been shaped in the same way as the modern shoreline and uplifted to elevations of 400 m (Fig. 1 and Supplementary Fig. 1). These palaeo-shorelines have been used to describe the progressive uplift and flexure synchronous with glacio-eustatic sea-level highstands^10^. Thus, their gradually deformed geometry may be used as a “palaeo-geodetic” strain marker, providing key observables to be reproduced by numerical modelling experiments that may help derive mechanical characteristics of the Corinth Rift’s evolution.

**Figure 2:**
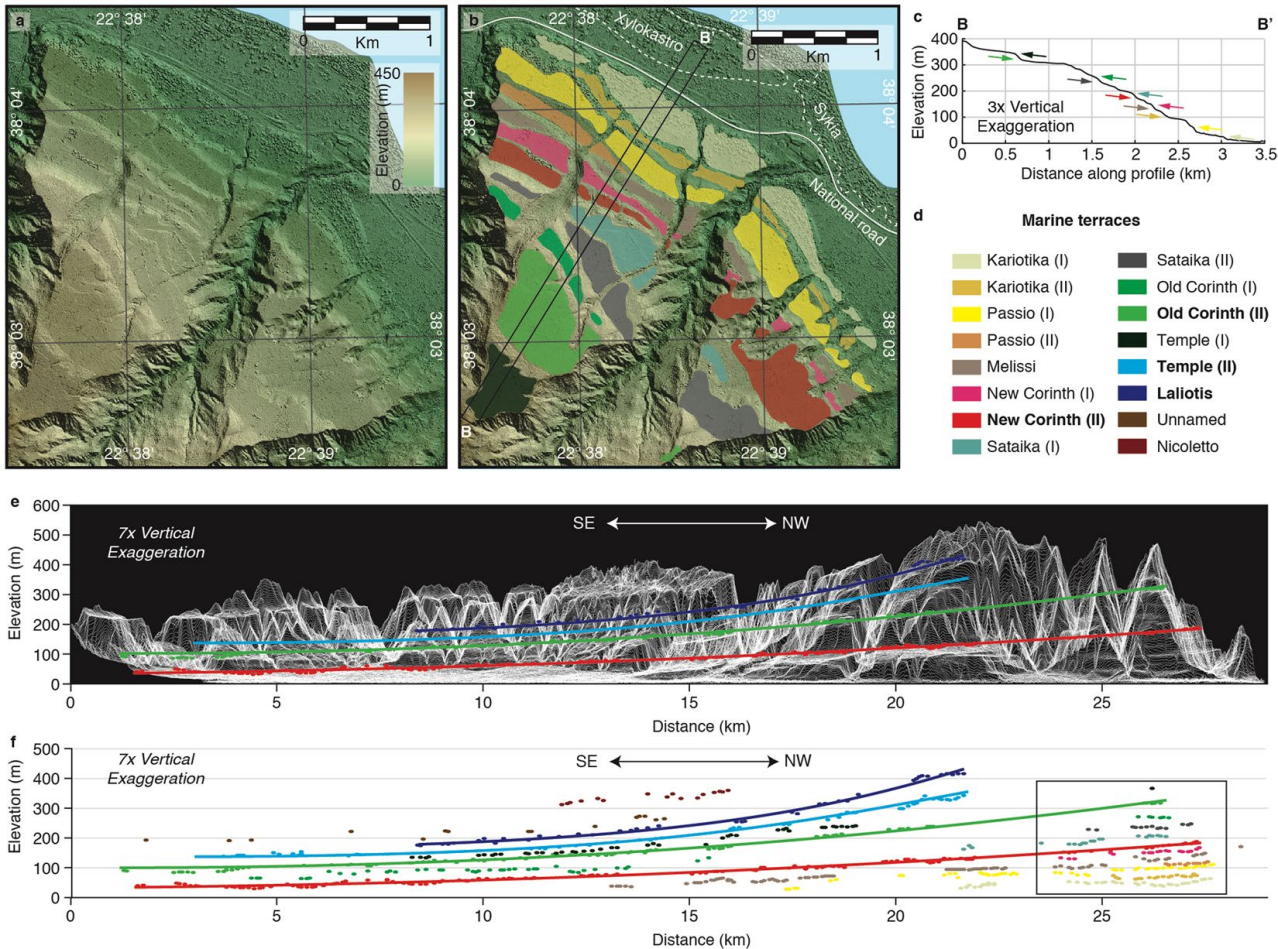
Detail of marine terraces on Pleiades DSM (**a**) Coloured hillshade DSM without interpretation, location given by inset in Fig. 1. (**b**) Same DSM with contouring of marine terraces. (**c**) Average swath topography through marine terraces levels, location given by inset in **b**. Arrows indicate differentiated terraces, colours indicated in **d** (**d**) Marine terrace legend, bold terraces are highlighted in (**e**,**f**) and Fig. 3. (**e**) Topography “view” parallel to the coast derived using stacked swath profiles with the shoreline angles and best fitting quadratic curves for the New Corinth (II), Old Corinth (II), Temple (II) and Laliotis terraces. (**f**) All determined shoreline angles along the same profile. Box shows location of **a** and **b**. Maps were made using MAPublisher version 9.8 (http://www.avenza.com/help/mapublisher/9.8/).

The Corinth terraces are generally composed of abrasion surfaces in soft Plio-Quaternary marls, sandstones and conglomerates, and are unconformably overlain by 2–6 m of erosion-resistant caprock consisting of well-cemented coastal deposits (Supplementary Fig. 2). In the area between Corinth and Xylokastro, we obtained a 2 m-resolution Digital Surface Model (DSM) from Pleiades satellite imagery. This DSM allows us to quantify the 3D terrace geometry with far greater detail than a typical open-source Digital Elevation Model (Fig. 2; Supplementary Fig. 3), and is available in the Supplementary Information.

**Figure 3:**
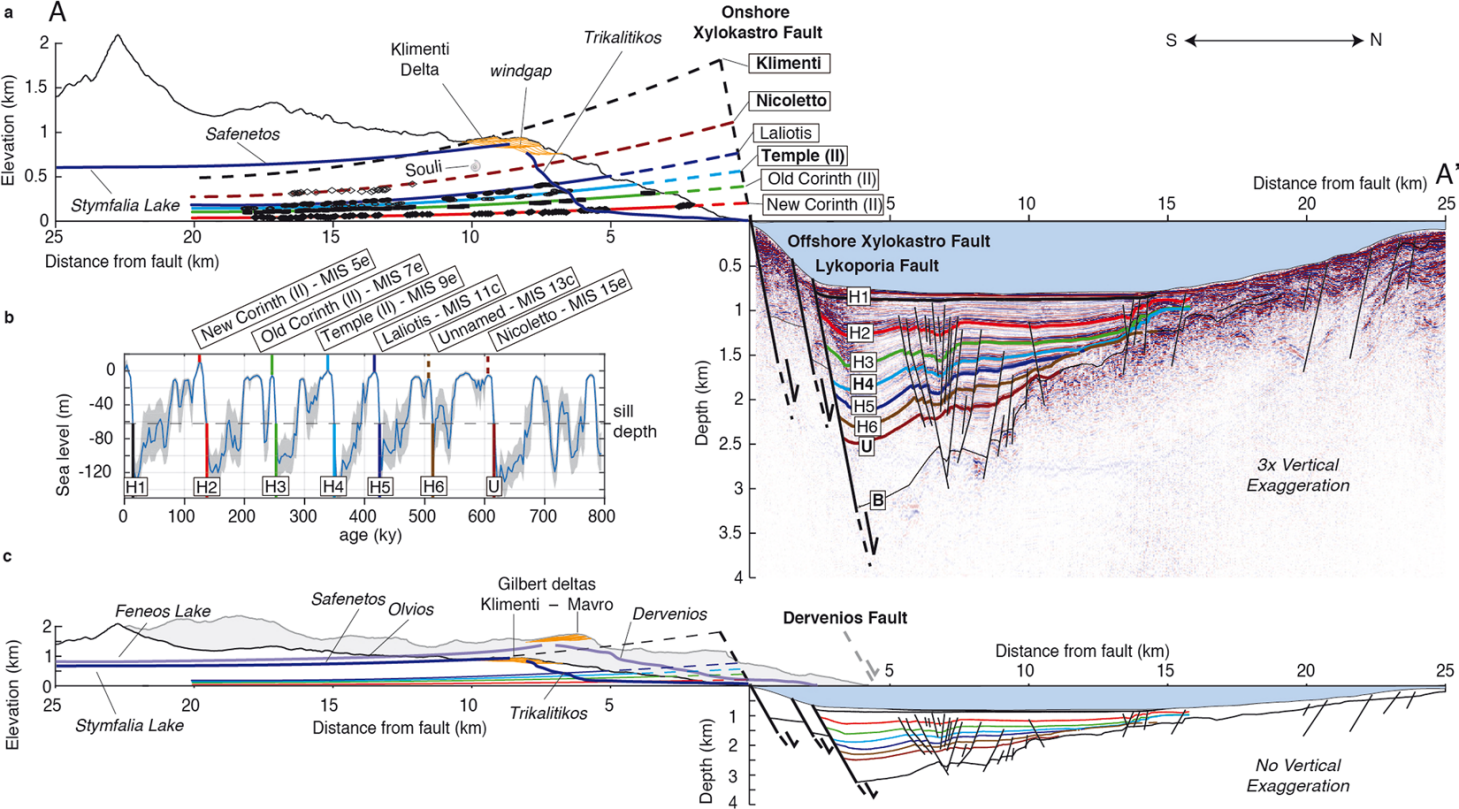
Combined on-offshore cross-section through Corinth Rift. (**a**) Cross-section with 3x vertical exaggeration, showing maximum topography of a 4-km wide swath profile across the Xylokastro terraces (Fig. 2a,b) and top of the Klimenti Delta (Fig. 1), shoreline angles of terraces assigned to major sea-level highstands with best-fitting quadratic curves and part of the Trikalitikos-Safenetos river system, all reprojected on line A-A’ of Fig. 1. Offshore seismic section is the interpretation of Nixon *et al*.^19^ on the depth-converted line L35 from Taylor *et al*.^20^ (**b**) Inferred ages of marine terrace levels and offshore seismic horizons plotted on the Pacific sea-level curve of Bates *et al*.^63^ (**c**) Main features of **a** without vertical exaggeration, and including the maximum topography of a 4-km wide swath profile parallel to A-A’ across the Mavro Delta.

Terraces are typically bounded inland by a palaeocliff. The intersection between terrace and palaeocliff, or shoreline angle (Supplementary Fig. 2), is considered the most appropriate datum of past sea-level position during the highstand they were formed^22,23^. We focus on the shoreline angles of terraces formed during major interglacial highstands (Fig. 3b), that are the widest and best preserved here^10^ and globally^24^, and have their corresponding glacio-eustatic sea-level less uncertain than lower interstadial highstands^25^. To determine which terraces correspond to interglacial highstands, we take into account available ages, and terrace width and preservation. To correlate undated terraces to interglacial highstands we assume approximately time-constant uplift rates, as is widely done in the analysis of marine terraces^23,24,26,27^, and has been suggested for sedimentation rates offshore^19^. We adopt the proposed terrace names by Armijo *et al*.^10^, and distinguish previously undescribed sub-levels with Roman numerals. Our high-resolution analysis allows us to detect both small (down to ~1 m) and strongly eroded cliffs, and hence more terrace sub-levels than previous studies. Sub-levels serve as guidelines for a precise spatial correlation across the whole flight of terraces, which increases the accuracy to determine the overall flexed terrace geometry and particularly the geometry of more eroded terrace levels older than the Old Corinth (II) (~240 ka). The shoreline angles determined for the wide and well-preserved terraces New Corinth (II) and Old Corinth (II) correlate with the two most recent interglacial highstands preceding the present-day one, Marine Isotope Stage (MIS) 5e (~124 ka) and MIS 7e (~240 ka) respectively (Fig. 3b). This age designation is supported by U/Th coral datings^28–30^ and IcPD dating of *Pecten*^31^. Although our shoreline angle elevations and terrace mapping are more accurate, this correlation is in essence similar to the interpretation of Armijo *et al*.^10^. However, our refined geometry and updated knowledge of glacio-eustatic sea-level variation^32^ leads us to propose correlation of the Temple (II) and Laliotis terraces to interglacial highstands MIS 9e (~326 ka) and MIS 11c (~409 ka) respectively, in better agreement than Armijo *et al*.^10^ with assumed time-constant uplift rates (Supplementary Fig. 4). Using the same assumption, terraces older than the designated Unnamed and Nicoletto would correspond to MIS 13e (~505 ka) and MIS 15c (~605 ka) respectively (Fig. 3b), but these old levels are significantly degraded and laterally discontinuous. After correlating main terrace levels to interglacial highstands, it logically follows that the secondary levels located in between those terraces should correspond to interstadial sea-level highstands lower than today’s sea level. Those interstadial levels are more numerous and better preserved at distances of ~2 km from the Xylokastro Fault (Fig. 2), where we derive the highest footwall uplift rate from the dated New and Old Corinth (II) terraces (~1.3 mm·yr^−1^; Supplementary Fig. 4).

The first order signal of the best-preserved terraces indicates a broad footwall flexure of at least ~20 km in relation to the Xylokastro on- and offshore faults and Lykoporia Fault (Figs 1 and 2). To estimate the long-term maximum footwall uplift rate at a hypothetical 0 km distance from the fault, we extrapolated the combined uplift rates of the New Corinth (II), Old Corinth (II), Temple (II) and Laliotis shoreline angles, obtaining an uplift rate of 1.6 ± 0.1 mm·yr^−1^, or 1.7 ± 0.1 mm·yr^−1^ if we exclude the slightly lower uplift rates of the New Corinth (II) terrace (Table 1; Supplementary Fig. 4).

**Table 1:** Uplift, subsidence, slip rate and U:S ratio.

Uplift rate		
	Max. UR (mm/yr) at Xylokastro Fault	Max. UR (mm/yr) at Lykoporia Fault
Inc. New Corinth (II)	1.6	1.9
Exc. New Corinth (II)	1.7	2.0
**Subsidence rate (palaeobathymetry 0 m, 610 ka)**		
Sediment decompaction	415 m	
	Max. SR (mm/yr) at Xylokastro Fault	Max. SR (mm/yr) at Lykoporia Fault
Exc. H2	4.0	3.7
**Subsidence rate (palaeobathymetry −800m, 610 ka)**		
Sediment decompaction	312 m	
	Max. SR (mm/yr) at Xylokastro Fault	Max. SR (mm/yr) at Lykoporia Fault
Exc. H2	2.4	2.2
**U:S ratio and slip rate**		
	High UR/Low SR	Low UR/High SR
U:S ratio	1:1.1	1:2.4
	Min. slip rate (mm/yr)	Max. slip rate (mm/yr)
40° fault	6.0	9.0
60° fault	4.5	6.7

Summary of main results, UR = Uplift Rate, SR = Subsidence Rate. More details are provided in the method section and Supplementary Fig. 4.

### Rift-Scale Cross-Section

The Rion and Acheloos sills^14^ in the western Gulf and the Corinth Isthmus in the eastern Gulf (Fig. 1), presently onshore, limited the water exchange during sea-level lowstands between the Gulf and the open sea over the past 600–700 ka^13^, resulting in alternating marine/lacustrine sedimentation found now both on- and offshore^15,16,33^. Long piston cores through the last lacustrine-marine transition ~13 ka^34,35^ have been correlated to distinct changes in seismic character within seismic profiles. On the basis of this seismic character change, several studies have interpreted the base horizon of deeper high amplitude packages as older lacustrine-marine transitions and correlated these to glacio-eustatic sea-level curves^12,19,36^ down to the basin-wide unconformity/seismic unit boundary U (Fig. 3). We used the most recent interpretation of seismic stratigraphy, faults and velocity model^19^ to depth-convert seismic line L35 of Taylor *et al*.^20^, and combined it with the onshore topography across the Klimenti Gilbert-type delta (hereafter Klimenti Delta) and the shoreline angles of major interglacial terrace levels (Fig. 3). The independently proposed timing of on- and offshore markers is similar. Small systematic differences of ~5–15 ka would thus correspond to lags between lacustrine to marine transitions and the maximum sea-level stands reached at the climax of interglacial periods (age differences between seismic horizons and terraces in Fig. 3b). We note that the uncertainty in these age differences is affected by both the choice of sea-level curve and evolution of sill depths through time.

Assuming that the highest Klimenti Delta foresets onshore and deepest sediments offshore mark the onset of slip along the Xylokastro and Lykoporia faults, the cross-section suggests that ~60–70% of the deformation associated with these faults has occurred over the past ~610 ka (our inferred age for the Nicoletto terrace and seismic horizon U^19^; Fig. 3). Before ~610 ka, detailed interpretation is hindered by the lack of well-developed marine terraces onshore and lack of clear seismic horizons offshore. The similarity in deformation pattern on both sides of the Xylokastro and Lykoporia fault system suggests that uplift and subsidence is a direct consequence of slip along those faults, and both sides can be directly compared over the past ~610 ka. The most elevated marine deposits in this area are found near Souli (Fig. 3), and have been tentatively dated as < 450 ka (last occurrence of *P. lacunosa*) using the nanoplankton assemblage of isolated samples^18^. We are sceptical about this age for two reasons: (1) The paleontological evidence is rather weak as the age is based on the absence rather than the presence of a nanoplankton species, and the samples are not part of a continuous stratigraphic section; (2) If correct, it would imply a threefold deceleration of uplift rate with respect to the more reliably dated New and Old Corinth terraces (from ~2.1 to ~0.7 mm·yr^−1^ at 10 km from the fault). Northward fault migration has occurred in the western Corinth Rift^37^, which could have locally lead to a sudden uplift rate deceleration, but the only major normal faults in this part of the Corinth Rift are the Xylokastro and Lykoporia faults, within a few kilometres distance from each other. Given the ~20 km uplift wavelength, simple migration of fault activity between these faults could not easily account for a threefold decrease in uplift rate at this distance from the fault. We do note the similarity in geometrical position between the highest marine deposits onshore and oldest marine incursion interpreted offshore (Souli/Nicoletto and horizon U; Fig. 3). This hints at continuous brackish/lacustrine conditions before ~610 ka, with less influence of glacio-eustatic sea-level cycles, and local climatic variations that are too small to produce lacustrine terraces and clear markers in offshore sedimentation.

Linear extrapolation using the uplift rate of the interpreted terraces suggests an age for the Klimenti Delta (Fig. 3) and initiation of the Xylokastro and/or Lykoporia faults of 1.0 ± 0.1 Ma. We note that given the uncertainty of extrapolating both the terrace geometry in space, and the uplift rates in time, the realistic uncertainty range of fault initiation is probably much higher than 0.1 Ma. Recent overview studies of the overall onshore stratigraphy along the gulf’s southern margin, which includes the Klimenti Delta, have estimated the age of this delta as Middle Pleistocene^37^, and as ~2–0.8 Ma^38^, with the onset of the Xylokastro Fault as old as ~2 Ma^38^. These ages are based on lateral correlations with biostratigraphically dated deltas further west, and U-Th dating of a tufa within the Klimenti Delta indicating an age older than ~600 ka^39^. Given these estimates, and as both linear extrapolation and lateral correlation approaches are difficult to (dis)prove without absolute ages, we keep a broad age range of ~2–0.9 Ma for the initiation of the Xylokastro and/or Lykoporia Faults, retaining the possibility that uplift rates have accelerated over the past ~2 Ma. The most elevated Gilbert-type delta in the gulf, the Mavro Delta further west (Fig. 1), has been uplifted by the onshore Xylokastro and Dervenios faults (Figs 1, 3). Comparing the Mavro and the Klimenti deltas, as well as their now inverted river drainage systems, we infer 1/3 more uplift for the Mavro than for the Klimenti delta (Fig. 3), and hence a slightly earlier onset of fault activity and/or higher average uplift rates.

**Figure 4:**
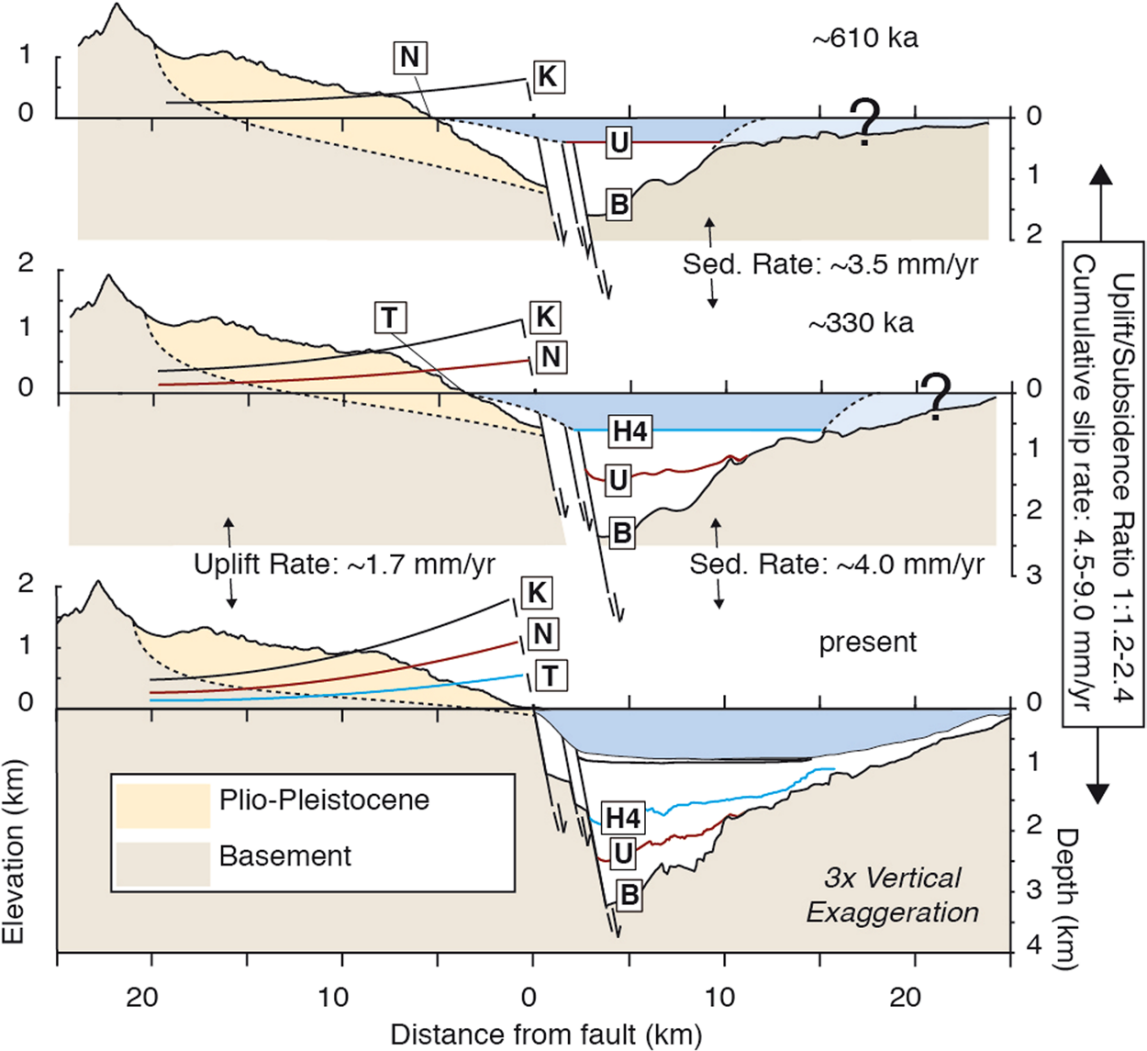
Schematic geologic restoration. The bold-labelled terraces and seismic horizons from Fig. 3 rotated back to horizontal, with the same rotation applied to the topo-bathymetry, accounting for sediment compaction.

We estimated the long-term slip rate and the uplift/subsidence (U:S) ratio by reconstructing the cross-section back to ~610 ka (Fig. 4). Palaeobathymetry in the gulf ~610 ka is unknown, and we assume a palaeobathymetry range between 800 m (current maximum water depth) and 0 m, as the two end-member scenarios in our reconstructions that correct for sediment compaction (see methods and Table 1). An important result is the constant or slightly decelerating uplift rates onshore comparing New Corinth (II) to older terraces, and acceleration of subsidence rates offshore comparing H2 to older horizons (Supplementary Fig. 4). This suggests a northward migration of fault activity (from Xylokastro to Lykoporia Fault, Fig. 3) affecting the most recent on-/offshore interglacial markers (~123–135 ka). Excluding those markers, and assuming most of the long-term deformation is related to the Xylokastro Fault, we estimate a long-term maximum subsidence rate of ~2.2–4 mm·yr^−1^, depending on the paleobathymetry ~610 ka (Table 1; Supplementary Fig. 4). Combining this with our long-term estimate of the maximum uplift rate from the marine terraces, and assuming the fault system is dipping 60° (Supplementary Fig. 5), we obtain a cumulative slip rate of 4.5–6.7 mm·yr^−1^ on the Xylokastro and Lykoporia faults and an U:S ratio of 1:1.1–2.4 (Table 1).

## Fault Modelling

**Figure 5:**
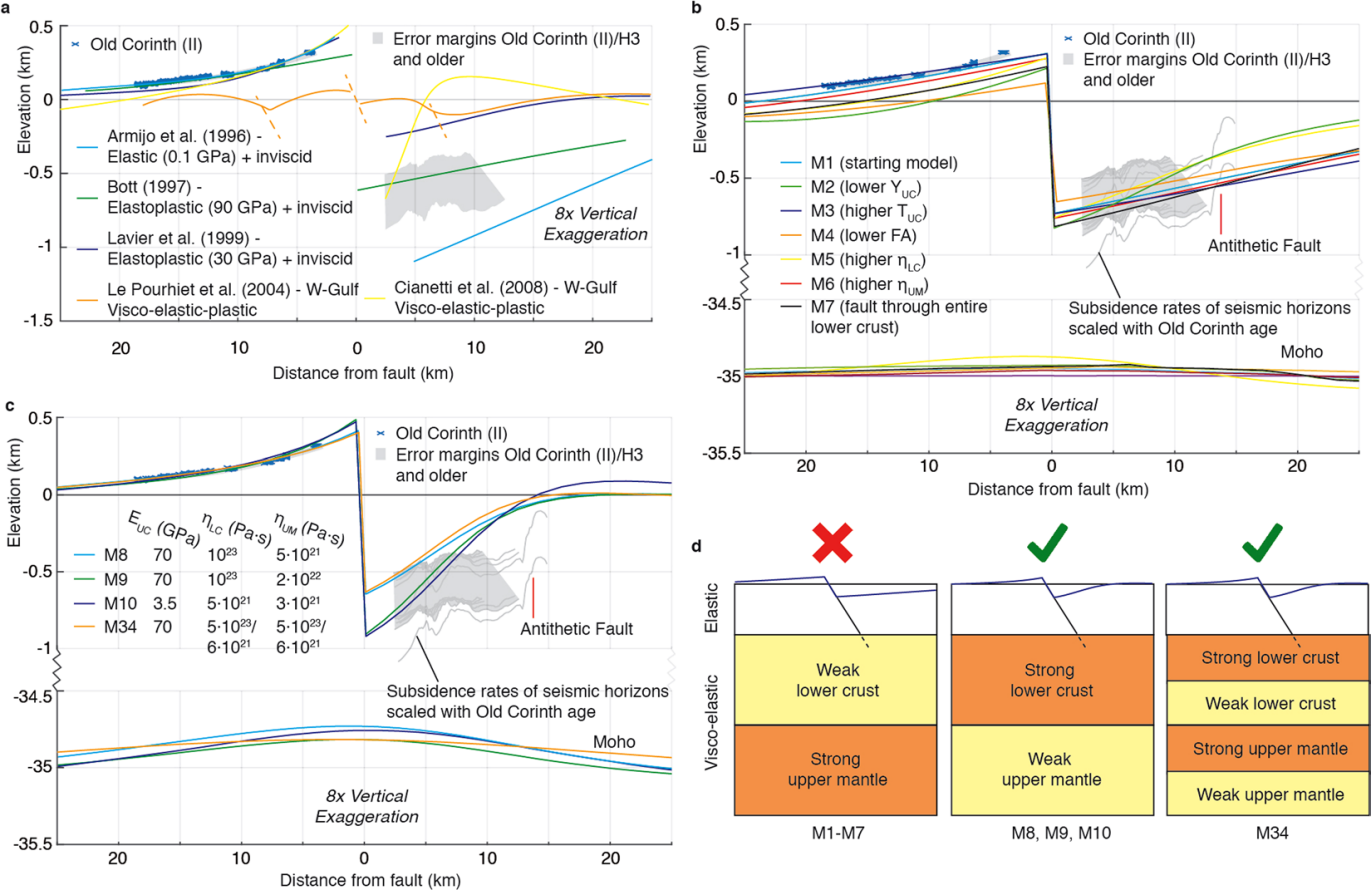
Fault modelling results. (**a**) Previous models within the Corinth Rift^10,44,45^ and two other models with inviscid lower crust^42,43^, all but Le Pourhiet *et al*.^44^ vertically scaled to the elevation of the Old Corinth (II) marine terrace (see methods) (**b**) Sensitivity tests for the different model parameters compared to the Old Corinth (II) terrace. Y_UC_ = Young’s Modulus of upper crust, T_UC_ = thickness of upper crust, FA = fault dip angle, η_LC_ = viscosity of lower crust, η_UM_ = viscosity of upper mantle. (**c**) Best-fitting models, which reproduce fault flexure by a relatively high viscosity lower crust (models 8 and 9) or an upper crust with relatively low Young’s Modulus (model 10). Model 8 has a slip rate of 4.5 mm·yr^−1^ and 0 mm·yr^−1^ of regional uplift rate, models 9 and 10 have a slip rate of 5.5 mm·yr^−1^ and 0.27 mm·yr^−1^ of regional uplift rate. Model 34 is the best-fitting model with 5 layers, in which the lower crust and upper mantle have the same viscosity, and is plotted here with a slip rate of 4.6 mm·yr^−1^ and 0.3 mm·yr^−1^ of regional uplift rate (**d**) Schematic representation of main modelling results.

The calculated U:S ratio is similar to the 1:1.1–2.2 estimate for the East Eliki Fault^40^ (Fig. 1) and values of ~1:1–2.5 for normal faults in the Basin and Range^41^, but at variance with previous numerical fault models for Corinth that predicted 2.7–3.5 times more subsidence than uplift for this fault system^10^. Other models with inviscid rheologies beneath the upper crust^42,43^ have reproduced the U:S ratio better, but do not adequately describe the flexure geometry observed in the rift (Fig. 5a). Modelling studies of deformation in the western Gulf considered multiple faults^44,45^ and resulted in flexure wavelengths dissimilar to those observed in our cross-section (Fig. 5a). Visco-elastic crustal-scale models^46^ illustrated the importance of fault geometry to reproduce the first order U:S pattern observed in the gulf, however these models did not account for crustal necking and neglected the role of the lithospheric mantle during flexure. All of the points mentioned above motivated an updated approach by incorporating visco-elastic lithosphere-scale models at high resolution. We follow the principle of King *et al*.^47^ that geological and geomorphic structures are the cumulative result of many earthquake cycles, and use a finite element model to solve for the surface displacements resulting from imposed normal slip on a planar fault in a simplified layered lithosphere made of either 3 or 5 layers. The fault plane runs through an elastic upper crust overlying a visco-elastic lower crust and upper mantle (Supplementary Fig. 7). We choose to reproduce the uplift and flexure pattern of the Old Corinth (II) terrace shoreline over 240 ka, because its deformed geometry is particularly well preserved and dated. We show the range of likely depths for the offshore markers (Fig. 5) defined by the estimated subsidence rates (Supplementary Fig. 4), but do not attempt to precisely reproduce them, due to the uncertainty in palaeobathymetry at the time of their formation, and the potential effects of secondary faulting offshore. Given the suspected northward fault migration discussed in the previous section, we assume most of the deformation for the Old Corinth (II) terrace (240 ka) has been caused by the on- and offshore Xylokastro faults. We use the approximately perpendicular profile A-A’ (Figs 1 and 3) as a reference section for our modelling, with the position of the onshore Xylokastro Fault as 0 m fault distance. We did not test the alternative possibility that the Lykoporia Fault instead of the Onshore Xylokastro Fault accommodated most deformation over ~240 ka. However, since only the terraces in the most NW part of the sequence could be affected by the Lykoporia Fault (Fig. 1) we do not expect this to significantly change our overall results.

In Fig. 5b and Supplementary Table 1 we show the parameters that we found have a major influence on the resulting deformation pattern of the 3-layer models. Compared to the reference model (M1), all these parameters influence the width of the uplifted zone, whereas all parameters but the upper crustal thickness influence the U:S ratio. The curvature of the footwall flexure is mainly influenced by the Young’s Modulus of the upper crust and the viscosity of the lower crust, increasing in flexure with lower and higher values, respectively. We show good fits to the data in Fig. 5c, using a 60° dipping fault as suggested from seismic data interpretation (Fig. 3; Supplementary Fig. 5), and a 10 km upper crustal thickness in agreement with the peak in microseismicity in this area of the Gulf (Supplementary Fig. 6). In our models we have used the range of possible slip rates from the previous section (4.5–6.7 mm·yr^−1^) and a regional uplift rate unrelated to rifting between 0–0.3 mm·yr^−1^ (see discussions in refs^10,48^). Assuming the long-term Young’s Modulus of the upper crust is comparable to typical values on coseismic timescales (see discussions in refs^10,47^), the lower crustal viscosity should be on the order of ~10^23^ Pa·s to reproduce the correct curvature of the terraces, and an upper mantle viscosity between 5·10^21^ and 2·10^22^ Pa·s is required to match reasonable slip rates and regional uplift rates (M8, M9 in Fig. 5c; Supplementary Fig. 7; Supplementary Table 2). Increasing the lower crustal viscosity by an order of magnitude has a similar effect on the curvature of the flexure as decreasing the upper crustal Young’s Modulus by an order of magnitude, but the latter has a stronger effect on the U:S ratio (M2 and M5; Fig. 5b). It is difficult to get good fits to the data with an upper mantle viscosity lower than ~3·10^21^ Pa·s and a Young’s Modulus and lower crustal viscosity lower than the values for M10 (Fig. 5c).

Previous studies on postseismic relaxation pointed out that models using only two homogeneous layers to represent the lower crust and upper mantle tend to result in a bias towards a relatively higher viscosity lower crust^49,50^. Therefore we also tested models in which we split both the lower crust and upper mantle in two separate layers, letting both the lower crustal and upper mantle viscosity decrease with depth (Supplementary Fig. 8, Supplementary Table 2). Unlike the 3-layer models, these 5-layer models have the lowest misfits with the uplift pattern for similar lower crustal and upper mantle viscosities (M34 in Fig. 5c, Supplementary Table 2).

Despite good fits to the uplift pattern, all the models in Fig. 5c show a mismatch of ~0–500 m with the subsidence pattern for distances >7.5 km from the Xylokastro Fault. We attribute the mismatch in reproducing the offshore pattern to the presence of antithetic faults ~15 km north of the Xylokastro Fault (Figs 3 and 5), which modify the flexure pattern. We tested if the models were sensitive to earthquake recurrence times and modelling with fixed or moving sidewalls (M11-M13 in Supplementary Fig. 7), both of which do not influence our results. Additional models with a 15 km upper crustal thickness, 50° fault, elasto-plastic upper crust and non-linear (powerlaw) visco-elastic lower crust are discussed in the Supplementary Information (Supplementary Fig. 7 and Supplementary Table 1), but also do not change our main results.

We did not include surface processes (footwall erosion and hanging-wall sedimentation) in our modelling approach, and acknowledge that these may play a role in both the flexural pattern and the surface U:S ratio. Earlier models explored the influence of surface processes (0–100% filling of basin) on 4 km thin elastic layers^47^, finding a minor increase in flexure wavelength (≤10%) and a decrease in U:S ratio (≤10%). Armijo *et al*.^10^ then used an adaptation of the same model with a very weak (E = 0.1 GPa) instead of thin elastic layer, and found that 10–25% of sediment erosion/deposition and 75–90% of water filling, estimated for the Gulf of Corinth, would have a negligible effect on the surface deformation pattern. In later work on a stronger (E = 50 GPa) and thicker (15 km) elastic plate, Maniatis *et al*.^51^ showed that there is no visible effect on the flexure wavelength/curvature and a decrease in U:S ratio of ≤20% as a result of surface processes (0–3 m^2^/a erosion/deposition). Considering these studies, the implementation of surface processes would, if anything, require a slightly stronger lower crust and/or weaker upper mantle within the models to fit our data. As such, we do not expect surface processes to affect our main modelling outcomes (Fig. 5d), especially given that the Corinth Gulf is underfilled and deposition occurs in limited depocenters localised in the faults’ hanging-wall^19^.

## Tectonic and Rheological Implications

Our revised slip rate of 4.5–6.7 mm·yr^−1^ for the combined Xylokastro/Lykoporia faults, based on both onshore and offshore data, and a 60° fault dip, is significantly lower than the previous estimate of 7.0–16 mm·yr^−1^ for this fault system by Armijo *et al*.^10^, and similar or slightly higher than rates of 3.5–5.5 mm·yr^−1^ proposed by Bell *et al*.^12^ The improvement to the slip rate estimate in comparison to the Armijo *et al*.^10^ rate mainly results from the incorporation of the offshore data constraining hanging wall subsidence into the flexural model and a different fault dip estimate. If we assume the fault system is dipping ~40° instead^20^, the cumulative slip rate would be 6.0–9.0 mm·yr^−1^ (Table 1), compatible with the previously estimated minimum rate of 7 mm·yr^−1^ but still with a much lower upper bound. The slightly lower estimate of Bell *et al*.^12^ results from not extrapolating the ~1.3 mm·yr^−1^ uplift rate from the terraces to the position of the fault(s) (Supplementary Fig. 4). These discrepancies emphasize the need to integrate on- and offshore data to estimate slip rates of major coastal fault systems, and thus for estimating potential earthquake recurrence and seismic hazards.

Compared to coseismic deformation, long-term patterns integrated over many earthquake cycles tend to have a lower U:S ratio and broader wavelength of deformation due to postseismic relaxation processes of the deeper layers^47^. The influence of the fault angle and upper crustal strength on this ratio has been pointed out by previous studies^10,47^ and our study demonstrates that the rheology of the lower crust and upper mantle also plays a major role in controlling the surface deformation pattern. Unlike those studies, we do not require the long-term upper crustal strength to be lower than the short-term strength, or the effective elastic thickness to be smaller than the depth of the seismogenic layer.

The best-fitting 3-layer models for the terraces (Fig. 5c) have a lower crustal viscosity that is 2–20 times higher than the upper mantle viscosity. The relatively localised Moho rise (Fig. 5c) in these models is a direct consequence of this viscosity contrast, and is in good agreement with local Moho geometry (Supplementary Fig. 6). Our results agree well with compilations of postseismic relaxation studies on 10^0^–10^3^ yr timescales that also show lower crustal viscosities to be generally higher than upper mantle viscosities in 3-layer models^52,53^, although ~1–3 orders of magnitude lower than our long-term viscosity estimates. Our tests on a 2.4 ka timescale (M28-M31 in Supplementary Fig. 7) fit within that context, showing that appropriate absolute viscosity values depend on the time period under consideration for relaxation, while the relative viscosity contrast remain immutable.

Within our 5-layer models (M34 in Fig. 5c, Supplementary Fig. 8), relatively localized Moho rise occurs across a viscosity contrast that is reversed with respect to 3-layer models, but produces equally good-fitting results (Supplementary Table 2). Our tests align with postseismic relaxation studies showing that 3-layer models are biased towards higher viscosity lower crust^49,50^, but on a considerably longer timescale (240 ka). Our 5-layer models achieve the best fits to the data with similar viscosity lower crust and upper mantle, and further increasing the amount of layers may also permit a good fit to the data with an upper mantle stronger than the lower crust. As a consequence, our models cannot unequivocally demonstrate that the lower crust in the Corinth Rift is stronger or weaker than the upper mantle. What the best-fitting 3- and 5-layer models do have in common is that most of the viscous relaxation takes place relatively deep, in the lower portion of the lower crust and/or upper mantle (Fig. 5d). Coming back to primary observations within our cross-section of the Corinth Rift, this is both intuitive and physically reasonable: viscous relaxation allows for higher U:S ratios with respect to coseismic elastic flexure, whereas its relatively deep occurrence allows for the topographic signal of coseismic elastic flexure to be well maintained at the surface throughout many earthquake cycles.

It was recently proposed that observed U:S ratios of 1:1–1:3 in normal fault systems evidence high-angle normal faulting, rather than low angle normal faulting^46^. This may be characteristic for all young, amagmatic rifts that are not close to breakup and have no optimally oriented pre-existing low-angle structures^46^. Our study shows that in addition to constraining the fault angle, the U:S ratio and flexure geometry resulting from normal faulting can be essential features to constrain rheological layering below such rifts. Since the few other long-term U:S ratio estimates for normal faults^40,41^ are similar to that we obtain in the Corinth Rift, and its record of flexure geometry is unparalleled worldwide, the topographic evolution in the Corinth Rift and its rheological layering may well typify rapid localised extension of continental lithosphere elsewhere.

## Methods

### Marine terrace analysis

To develop the DSM, we obtained tri-stereo Pleiades satellite images of 0.5 m-resolution covering the terrace sequence between Xylokastro and Corinth. The open-source software MicMac^54,55^ was used to create tie-points, orientate the images and calculate a 0.5 m-resolution DSM, using ground control points at 0 m elevation for several locations along the coastline. To reduce the topographic effects of vegetation, crops and man-made structures, the DSM was downsampled to 2 m resolution (Fig. 2).

Mapping of the terraces (Figs 1 and 2 and Supplementary Fig. 1) was done semi-automatically using the surface classification model of Bowles and Cowgill^26^, which combines the slope and roughness linearly to detect relatively low-slope smooth surfaces. Contours around those surfaces were drawn manually using a combination of satellite imagery, slope maps and hillshade images of the DSM. The slope of the Holocene seacliff was measured at 48 locations and its value ±1σ was used to estimate the horizontal and vertical position of the shoreline angles for ~700 palaeocliffs with TerraceM^56,57^ (Supplementary Figs 2 and 9). To reduce the influence of fluvial and gravitational erosion, we used the maximum topography of 100 m-wide swath profiles perpendicular to the cliffs, the size preferred by Jara-Muñoz *et al*.^57^. All swath profile and shoreline angle locations are included as supplementary Google Earth and ESRI Shapefile data files. The terraces were correlated laterally using satellite imagery, mapview and profile view of shoreline angles in combination with a N130°E coast-parallel swath stack^58^ of 500 average elevations of swath profiles (Fig. 2d).

The uplift rate *U* for individual shoreline angles was calculated using U = (H_T_ − H_SL_)/T, where H_T_ is the present elevation above the modern mean sea-level, H_SL_ is the eustatic sea-level elevation for the time interval of terrace formation and T is the age of terrace formation. Following Gallen *et al*.^59^, standard errors SE were calculated using:

$$SE{(u)}^{2}={u}^{2}((\frac{{{\sigma }_{H}}^{2}}{{({H}_{T}-{H}_{SL})}^{2}})+(\frac{{{\sigma }_{T}}^{2}}{{T}^{2}}))$$

where σ_H_ is the combined uncertainty of shoreline angle elevation and eustatic sea-level correction, and σ_T_ is the uncertainty in age of terrace formation. For the eustatic sea-level highstands MIS 5e, MIS 7e, MIS 9e and MIS 11c, correlated to the New Corinth (II), Old Corinth (II), Temple (II) and Laliotis terraces, we used the eustatic highstand age uncertainty of 123.5 ± 8.5 ka, 240 ± 6 ka, 326 ± 9 ka and 409 ± 16 ka^60^ to represent the uncertainty in age of terrace formation. As eustatic sea-level corrections for those same highstands we used 5.5 ± 3.5 m, 0.5 ± 3.5 m, 2.5 ± 5.5 m and 5 ± 8 m^32^, and added these uncertainties to the uncertainties calculated for each individual shoreline angle (Supplementary Figs 2 and 9). Although the error bars of the older terraces are smaller due to the smaller influence of age uncertainty (see equation above), we note that the actual uncertainty of the rates derived from those terraces is much higher since those levels have not been directly dated. The uplift rate at the onshore Xylokastro Fault was estimated by combining the New Corinth (II), Old Corinth (II), Temple (II) and Laliotis uplift rates and extrapolating a best fitting quadratic curve with MATLAB (Supplementary Fig. 4). A critical χ^2^ test was done to confirm that the residuals follow a Gaussian distribution and the uplift rate dataset is well described by the curve^61^, which was the case when excluding the New Corinth (II) terrace, but not when including that terrace. Within this test we excluded the New Corinth (II) datapoints between 13 and 18 km distance from the fault, since their elevation appears to be disturbed by sedimentary processes on the Vokha plain (Fig. 1), particularly around rivers.

### Constructing cross-section and evolution model

We depth-converted the multi-channel seismic section L35^20^ using the velocity model of Taylor *et al*.^20^, and adopted the interpretation of faults and seismic horizons from Nixon *et al*.^19^ (Supplementary Fig. 5). The shoreline angles were reprojected on a profile of the same orientation as the seismic section, approximately perpendicular to the on- and offshore Xylokastro Fault (Figs 1 and 3). To combine the terraces with topsets of the overlying Klimenti Delta the maximum elevation of a 4-km wide, N025E oriented swath profile was also reprojected along the same line (Fig. 1). The river profile of the inverted Safenatos River and the windgap-connected trunk of the Trikalitikos river were merged together, and horizontally scaled to have the windgap at the correct location within the cross-section and the river outlet at the coastline. Best-fitting quadratic curves for the New Corinth (II), Old Corinth (II), Temple (II) and Laliotis terraces were extracted with MATLAB. The Laliotis curve, assuming an age of 409 ka and a eustatic sea-level correction of + 5 m, was extrapolated linearly to estimate total uplift at 605 ka and 1050 ka, approximately corresponding to the sea-level highstand following the oldest marine incursion interpreted offshore (Fig. 3b) and the age to match the position of the Klimenti Delta overlying the terrace sequence (Fig. 3a). The sill depth is chosen at 62 m^62^, and for simplicity chosen as constant through time. Given the fast rate of sea-level rise before major interglacial highstands, uncertainty in sill depth does not change the age of the interpreted offshore horizons much, nor does the depth-uncertainty in the sea-level curve. We chose to display the sea-level curve of Bates *et al*.^63^ in Fig. 3, since it is the most recent curve that we are aware of covering >610 ka that is accounting for global observations of uplifted palaeoshorelines, and use their equatorial Pacific curve since they use it as reference curve. For the background topography comprising the Mavro Delta (Fig. 3c) we used a 4-km wide swath profile along the same orientation as A-A’.

In the evolution model (Fig. 4) the palaeodepth of the Corinth Gulf at 605 ka was chosen at 400 m as an average of two end-member scenarios at which seismic horizon U would represent the sea-level at 0 m, or the local sea bottom at its present-day depth of ~800 m. Sea/lake deepening was assumed to be constant between 605 ka and present, and sediments were decompacted using a porosity-depth relationship for calcareous sediments from Nixon *et al*.^19^, based on experimental data^64^. See Nixon *et al*.^19^ for a full discussion of the decompaction parameters. To calculate a subsidence rate over the past ~610 ka we estimated the subsidence of seismic horizon U, taking end-member scenarios of 0 and 800 m palaeo sea/lake depth into account. We used the current depth of seismic horizon U of ~2480 m depth, substracting 0–800 m for the sea/lake palaeodepth and 415–312 m due to compaction of the sediments below the U horizon. The same principle was applied for every individual horizon in Supplementary Fig. 4, and used for the error margins in Fig. 5 and Supplementary Fig. 7. We used the equatorial pacific sea-level curve of Bates *et al*.^63^ as well as the sea-level curve of Spratt and Lisiecki^65^ to determine the timing of the horizons, noting that for the subsidence rate calculations the uncertainty in used sea-level curve affects the outcome much less than the paleobathymetry. Since reconstruction of the maximum swath profile topography from Fig. 3 should be relatively insensitive to river incision, we did not take into account onshore erosion processes in Fig. 4.

### Fault modelling

For the fault modelling we used PyLith^66^, an open-source finite element code for dynamic and quasi-static simulations of crustal deformation. We used a starting model with a 10 km upper crustal thickness, adopting the peak in microseismicity depth (Supplementary Fig. 6) around the cross-section of Fig. 3, and a 35 km crustal thickness following Moho depth estimates from Ps receiver functions^67^ and tomographic inversion of PmP reflection times^68^. Listric and biplanar fault geometries were excluded from our models, since they are not expected to give the significant footwall uplift that our data suggests^46^. For model simplicity we exclude erosion and sedimentation processes, to which previous numerical models with much lower upper crustal Young Modulus were relatively insensitive^10^. We used 2.5 m normal slip earthquakes with a recurrence time of 500 years, following our range of estimated long-term slip rates, and roughly in agreement with the recurrence times for major earthquakes inferred from offshore palaeoseismology^69^. The models have uniform slip until the base of the upper crust, linearly decreasing to 0 m slip between 10 and 12 km depth to avoid extreme boundary effects at the fault tip. Sensitivity tests suggest the models are insensitive to the recurrence time if the slip rate is the same, and the ground surface pattern for different slip rates can be approximated by linear inter- or extrapolation of the displacement vector after the model run (Supplementary Fig. 7). For the models with moving walls we applied a 1.25 mm·yr^−1^ horizontal velocity for both walls to ensure all extension in the model is taken up by the 5 mm·yr^−1^ slip along the fault. We applied an upward velocity of 0.03 mm·yr^−1^ to isostatically compensate for the thinning of the crust. For the models with an elastoplastic upper crust (Supplementary Fig. 7) we used the plastic parameters from Cianetti *et al*.^45^. For the models with a non-linear (powerlaw) viscoelastic lower crust we used the quartz flow law from Gleason and Tullis^70^. For the five-layer models we used similar parameters to the starting model, and systematically varied viscosities between 3·10^21^ and 5·10^23^ Pa·s (Supplementary Fig. 8, Supplementary Table 2), which is a similar range to our best fitting 3-layer models (Fig. 5c). In all model runs we included gravitational body forces and used a finite strain formulation.

For the comparison with previous numerical models (Fig. 5a) we vertically rescaled the deformation pattern of selected models in those studies to approximately match the Old Corinth (II) terraces. From Armijo *et al*.^10^ this is their figure 23, from Bott *et al*.^42^ this is the model in their figure 7b with an appropriate U:S ratio, from Lavier *et al*.^43^ this is the model in their Fig. 2 (bottom) and from Cianetti *et al*.^45^ this is the model in their Fig. 3b with an appropriate U:S ratio. Le Pourhiet *et al*.^44^ argue in their text for ~2.0 mm·yr-1 of regional uplift, and given the complicated multi-fault deformation pattern in their preferred model in their Figure 8d we did not apply this correction, nor did we scale it vertically to the Old Corinth (II) terraces.

## Electronic supplementary material

Supplementary Information

## Data Availability

The Pleiades satellite imagery was obtained through the ISIS and Tosca programs of the Centre National d’Etudes Spatiales (CNES, France) under an academic license and is not for open distribution. On request, we’ll provide the DSM calculated from this imagery to any academic researcher who gets approval from CNES (contact isis-pleiades@cnes.fr for quoting this paper, and with lacassin@ipgp.fr in copy). We do share a georeferenced hillshade image and slope map of the 2 m-resolution Digital Surface Model that was developed from Pleiades satellite imagery. This image can be retrieved with these links: https://figshare.com/s/05d6610458391e9da3d7(hillshade image) https://figshare.com/s/a50519854408656e2532(slope map). The other data that support the findings of this study are available within the publication, referenced studies and/or from the corresponding author on request.
