# Supplementary material for: Lithospheric flexure and rheology determined by climate cycle markers in the Corinth Rift
Source: Sci Rep. 2019 Mar 7;9:4260. doi: 10.1038/s41598-018-36377-1 (PMC6403285; doi:10.1038/s41598-018-36377-1)
Supplement: Supplementary file 1 — Supplementary Information [file 41598_2018_36377_MOESM1_ESM.pdf]

Supplementary Information

(Lithospheric flexure and rheology determined by climate cycle markers in the Corinth Rift)

Gino de Gelder[1*], David Fernández-Blanco[1], Daniel Melnick[2,3], Guillaume Duclaux[4], Rebecca E. Bell[5], Julius Jara-Muñoz[2], Rolando Armijo[1] and Robin Lacassin[1])

This file includes 8 supplementary figures, and 2 supplementary table. Supplementary Figure 9 is a data repository and can be retrieved with this link:

https://doi.org/10.6084/m9.figshare.5406358

Additionally, we share a georeferenced hillshade image and slope map of the 2 m-resolution Digital Surface Model that was developed from Pleiades satellite imagery, and formed the basis for marine terrace analysis. This image can be retrieved with these links:

https://doi.org/10.6084/m9.figshare.5406547(hillshade image)

https://doi.org/10.6084/m9.figshare.5406481(slope map)

Supplementary text on numerical modelling

Supplementary Fig. 7 shows additional modelling results that serve to demonstrate that our main conclusions for 3-layer models are relatively insensitive to the chosen model set-up and parameters, whereas Supplementary Fig. 8 shows our 5-layer modelling results.

In Supplementary Fig. 7b we show with M11 that changing the recurrence time, while keeping the same slip rate, results in a visually indistinguishable deformation pattern. M12 shows that the effect of changing the slip rate can be well approximated by correcting the final displacement pattern, as is done in our tests to systematically find the slip rates and regional uplift rates with the lowest misfits to the data (Supplementary Fig. 7f, 8e). In Supplementary Fig.

7c we show that our choice of a model set-up with fixed sidewalls does not influence the final deformation pattern significantly. M14 in Supplementary Fig. 7d shows that using two orders of magnitude lower viscosities with respect to M8 results in an unrealistic topographic evolution after >7 ka of running the model.

In Supplementary Figs. 7e and 7f we show that with a lower crustal viscosity of 1023, necessary to preserve the observed curvature of the elastic flexure signal, lower upper mantle viscosities than $5{\cdot}10^{21}$ Pa·s (M15) or higher viscosities than $2{\cdot}10^{22}$ Pa·s (M16, M17) result in too much and too little uplift respectively. Models with a 15km thick upper crust (Supplementary Fig. 7g) do not reproduce as much flexure of the curvature as M8 and M9 for the same upper crustal Young's Modulus and viscosity values (M18, M19), but with a strongly decreased upper crustal Young's Modulus (M20) we obtain good fits. This trade-off between upper crustal Young's Modulus and layer thickness has been proposed in several earlier studies (refs. 10,44 and references therein). Models with a lower fault angle (Supplementary Fig. 7h), require slightly lower upper mantle viscosities (M21, M22) with respect to M8 and M9 (Supplementary Fig. 7e) to obtain similarly good fits to the data.

The effects of introducing an elastoplastic upper crust instead of a purely elastic upper crust are presented in Supplementary Fig. 7i, and indicate that this does not change the uplift pattern much. Models with an elastoplastic upper crust and non-linear (powerlaw) viscoelastic lower crust (Supplementary Fig. 7j) produce a realistic surface deformation pattern on a ~30 ka timescale, but on the long term they produce an unrealistically low U:S ratio.

We also tested how to reproduce the same uplift pattern on a shorter timescale (2.4 ka instead of 240 ka; Supplementary Fig. 7l), and find that this requires ~100 times lower viscosities for the lower crust and upper mantle (M28-M30). Using the same high viscosities as M8 on a 2.4 ka timescale results in too little uplift (M31), whereas using the same low viscosities as M28 for a 240 ka timescale results in an unrealistic topographic evolution after ~7 ka (M14 in Supplementary Fig. 7d).

Our 5-layer models are presented in Supplementary Fig. 8. With a similar viscosity lower crust and upper mantle we find the lowest misfits for the models with basal lower crustal and basal upper mantle viscosities of $6 \cdot 10^{21}$ Pa·s (M34, M35 in Supplementary Fig. 8b,e Supplementary Table 2), whereas models with lower and higher viscosities produce too much (M32, M33) and too little uplift (M36, M37). For the models with a slightly higher viscosity lower crust than upper mantle (Supplementary Fig. 8c), we find low misfits for the models in which the difference between lower crust and upper mantle viscosity is relatively small (M42, M43 in Supplementary Fig. 8c,e Supplementary Table 2). Similarly, for the models with a slightly lower viscosity lower crust than upper mantle (Supplementary Fig. 8d), we also find low misfits for the models in which the difference between lower crust and upper mantle viscosity is relatively small (M46, M50 in Supplementary Fig. 8d,e Supplementary Table 2). Overall, this indicates that we require similar viscosities for lower crust and upper mantle to reproduce the deformation pattern with the 5-layer models.

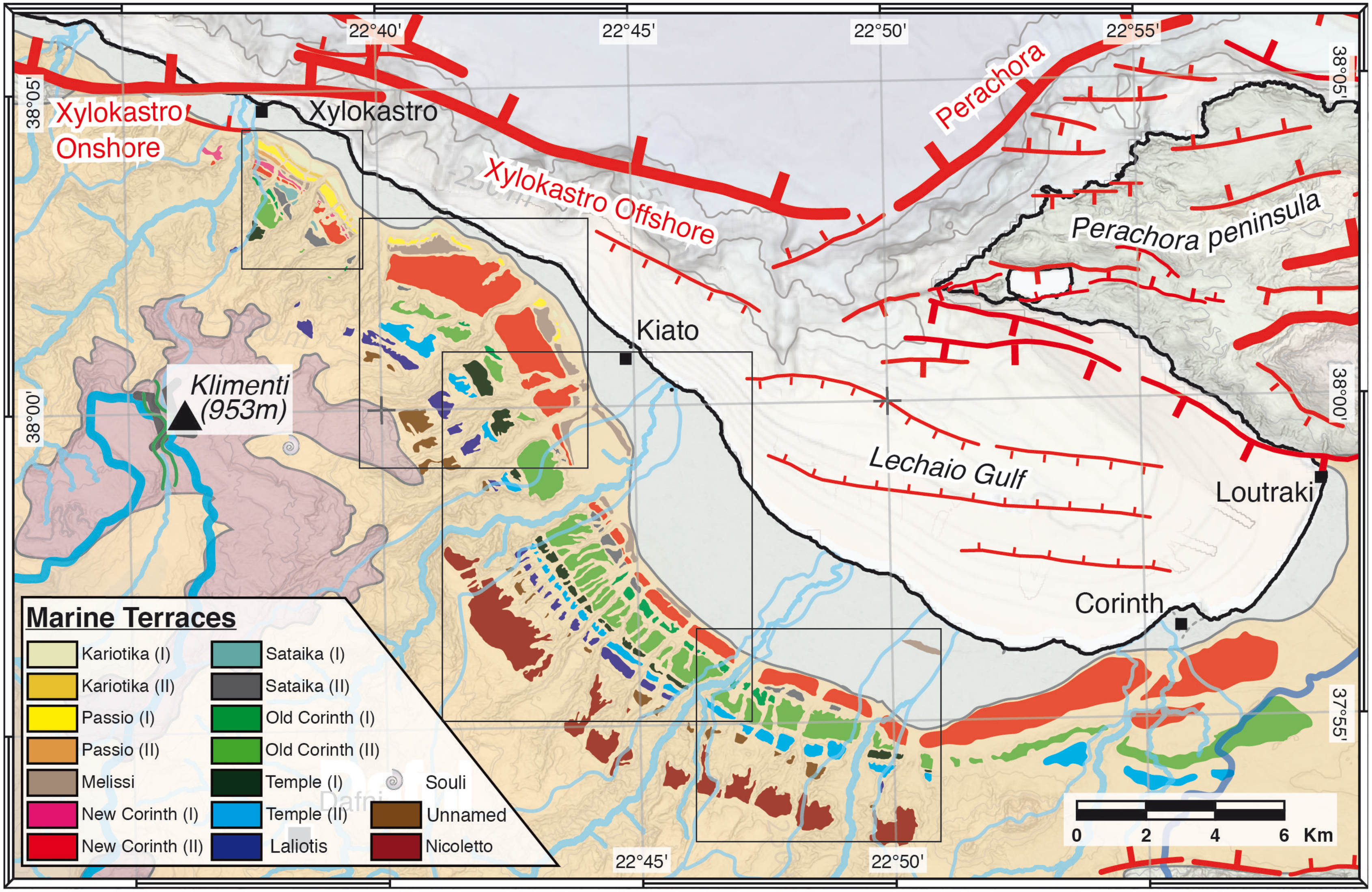
22°40'
22°45'
22°50'
22°55'
38°05'
38°00'
37°55'
Xylokastro Onshore
Xylokastro
Xylokastro Offshore
Perachora
Perachora peninsula
Kiato
Klimenti (953m)
Lechaio Gulf
Loutraki
Corinth
Marine Terraces
Kariotika (I)
Kariotika (II)
Passio (I)
Passio (II)
Melissi
New Corinth (I)
New Corinth (II)
Sataika (I)
Sataika (II)
Old Corinth (I)
Old Corinth (II)
Temple (I)
Temple (II)
Laliotis
Souli
Unnamed
Nicoletto
0 2 4 6 Km

Supplementary Figure 1 (previous page): **Marine terraces between Xylokastro and Corinth.** Based on detailed mapping with Pleiades DSM, with terrace names modified from Armijo et al.[10] (see text). Boxes indicate location of maps in Supplementary Fig. 8. Map was made using MAPublisher version 9.8 (http://www.avenza.com/help/mapublisher/9.8/).

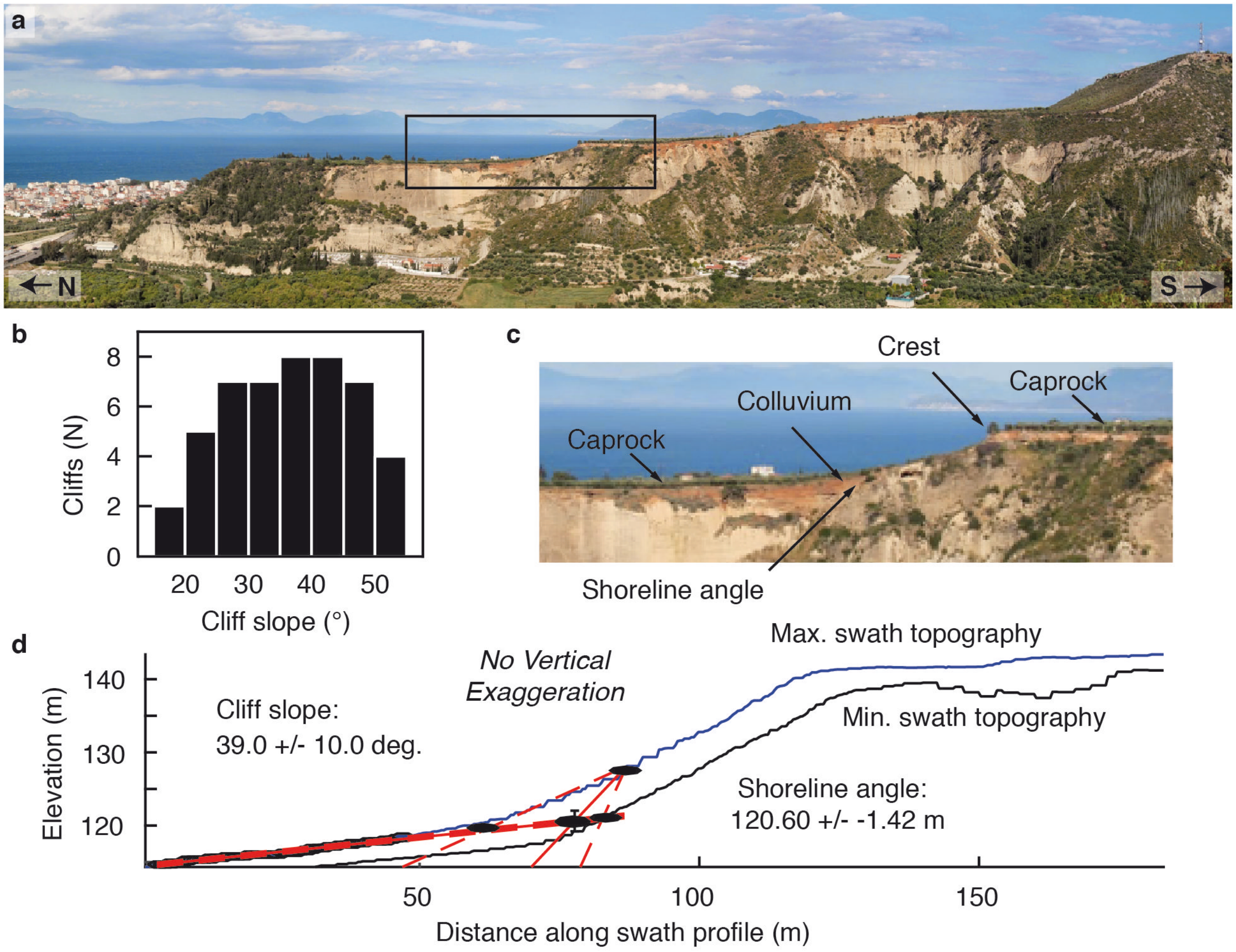

Supplementary Figure 2: Shoreline angle determination. (a) View of terraces near Xylokastro **(b)** Histogram of Holocene cliff slope measurements **(c)** Detail of terrace morphology from inset in **a** **(d)** Example of TerraceM shoreline angle analysis

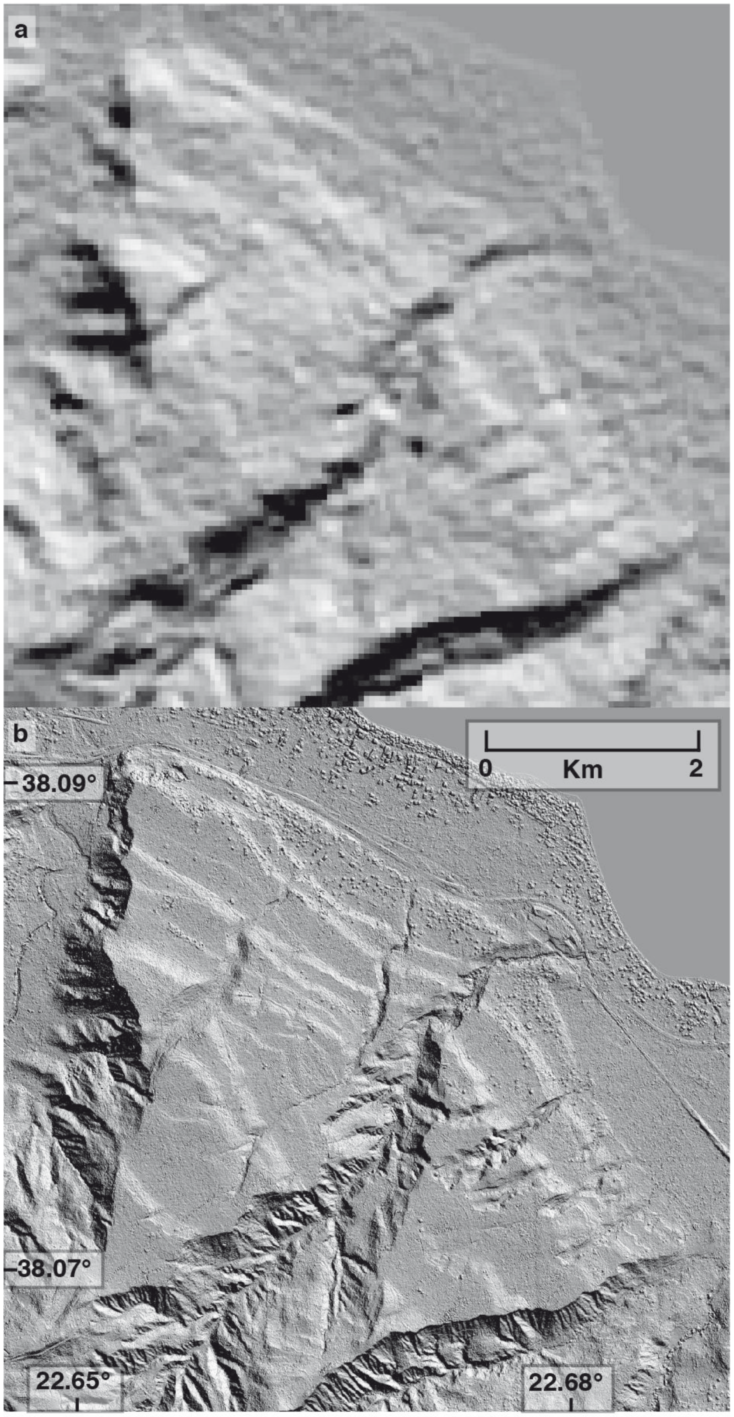

Supplementary Figure 3: Digital Elevation Model comparison. Hillshade images from **(a)** an ASTER DEM of 30m resolution and **(b)** a Pleiades DSM of 2m resolution.

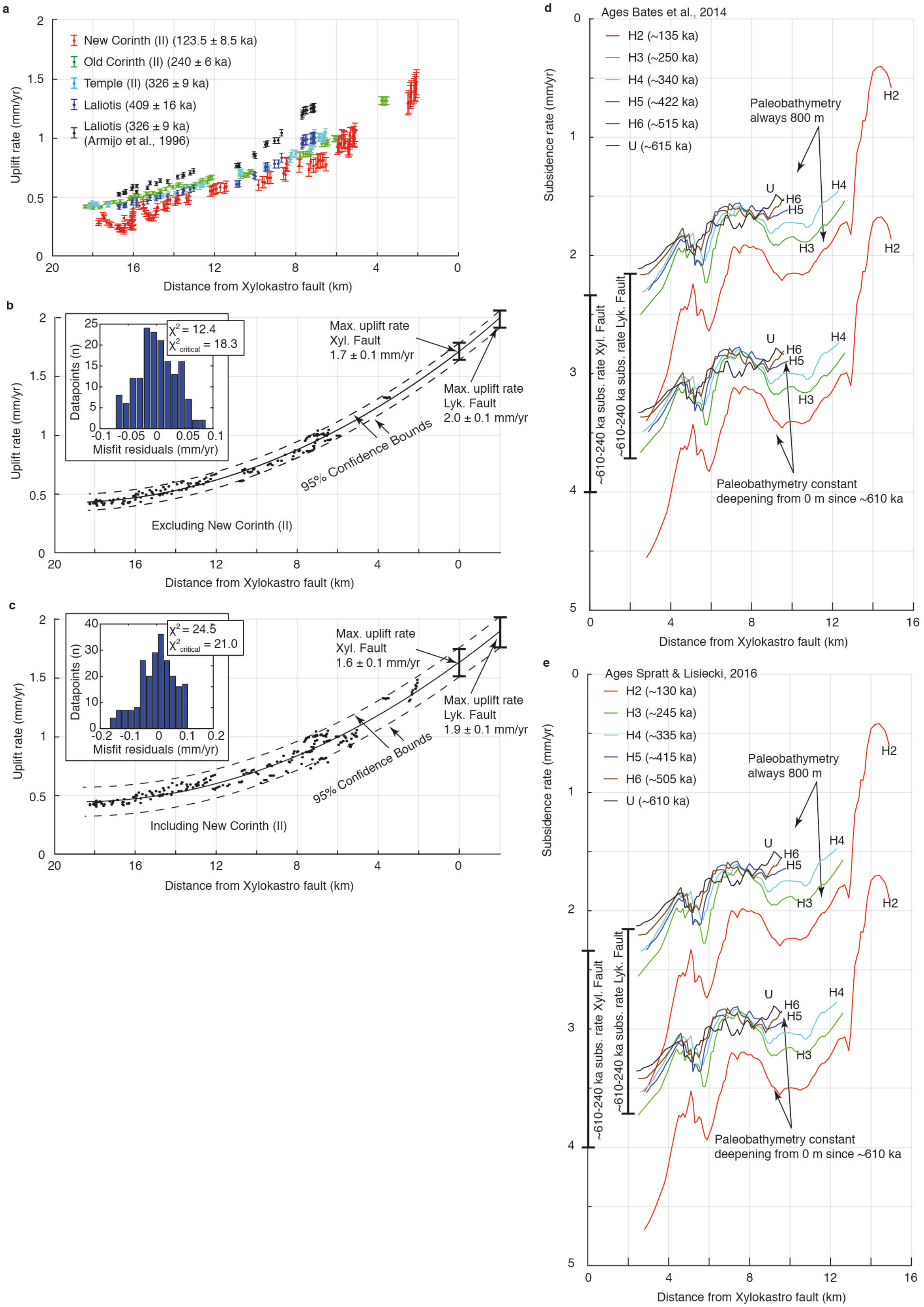
a
New Corinth (II) (123.5 ± 8.5 ka)
Old Corinth (II) (240 ± 6 ka)
Temple (II) (326 ± 9 ka)
Laliotis (409 ± 16 ka)
Laliotis (326 ± 9 ka)
(Armijo et al., 1996)
Uplift rate (mm/yr)
Distance from Xylokastro fault (km)
b
Datapoints (n)
Misfit residuals (mm/yr)
χ² = 12.4
χ²critical = 18.3
Max. uplift rate
Xyl. Fault
1.7 ± 0.1 mm/yr
Max. uplift rate
Lyk. Fault
2.0 ± 0.1 mm/yr
95% Confidence Bounds
Excluding New Corinth (II)
Uplift rate (mm/yr)
Distance from Xylokastro fault (km)
c
Datapoints (n)
Misfit residuals (mm/yr)
χ² = 24.5
χ²critical = 21.0
Max. uplift rate
Xyl. Fault
1.6 ± 0.1 mm/yr
Max. uplift rate
Lyk. Fault
1.9 ± 0.1 mm/yr
95% Confidence Bounds
Including New Corinth (II)
Uplift rate (mm/yr)
Distance from Xylokastro fault (km)
d
Ages Bates et al., 2014
H2 (~135 ka)
H3 (~250 ka)
H4 (~340 ka)
H5 (~422 ka)
H6 (~515 ka)
U (~615 ka)
Paleobathymetry
always 800 m
Paleobathymetry constant
deepening from 0 m since ~610 ka
~610-240 ka subs. rate Xyl. Fault
~610-240 ka subs. rate Lyk. Fault
Subsidence rate (mm/yr)
Distance from Xylokastro fault (km)
e
Ages Spratt & Lisiecki, 2016
H2 (~130 ka)
H3 (~245 ka)
H4 (~335 ka)
H5 (~415 ka)
H6 (~505 ka)
U (~610 ka)
Paleobathymetry
always 800 m
Paleobathymetry constant
deepening from 0 m since ~610 ka
~610-240 ka subs. rate Xyl. Fault
~610-240 ka subs. rate Lyk. Fault
Subsidence rate (mm/yr)
Distance from Xylokastro fault (km)

Supplementary Figure 4 (previous page): Uplift/subsidence rates. (a) Estimated uplift rates for selected shoreline angles from Fig. 2f **(b)** Old Corinth (II), Temple (II) and Laliotis uplift rates grouped together and best fitting quadratic curve, including 95% confidence bounds, extrapolated to estimate uplift rate near fault. Inset shows histogram of residuals and values for critical χ^2 test. **(c)** Same as **c**, but including the New Corinth (II) terrace **(d)** Estimated subsidence rates for seismic horizons in Fig. 3, using ages derived from the Bates et al.[63] Equatorial Pacific curve and **(e)** The same but with ages derived from the Spratt and Lisiecki[65] sea-level curve.

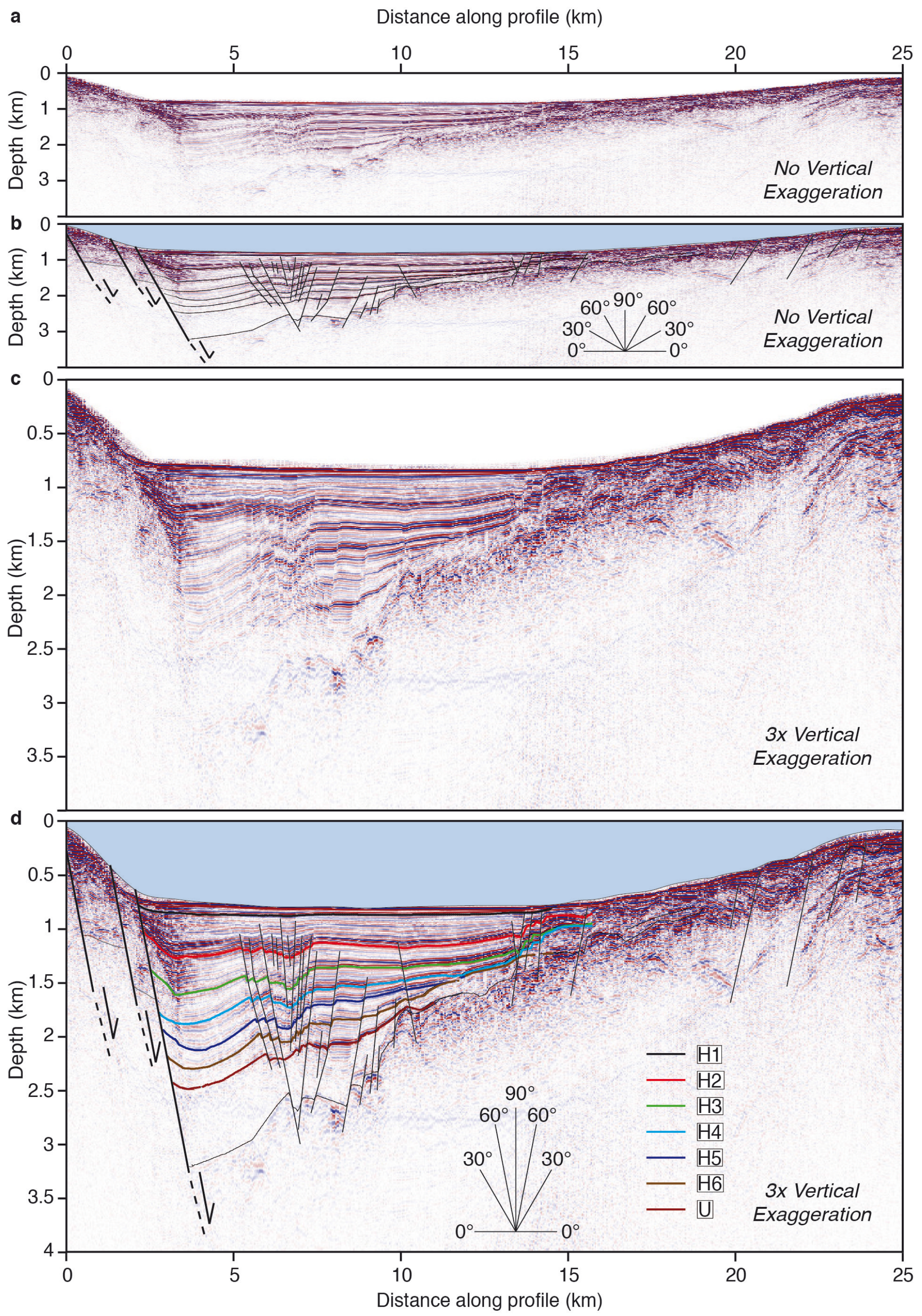

Supplementary Figure 5: Converted offshore seismic section of line L35[20]. (a) Without interpretation and vertical exaggeration **(b)** With interpretation from Nixon et al.[19] and without vertical exaggeration **(c)** Without interpretation and with 3x vertical exaggeration **(d)** With interpretation from Nixon et al.[19] and 3x vertical exaggeration.

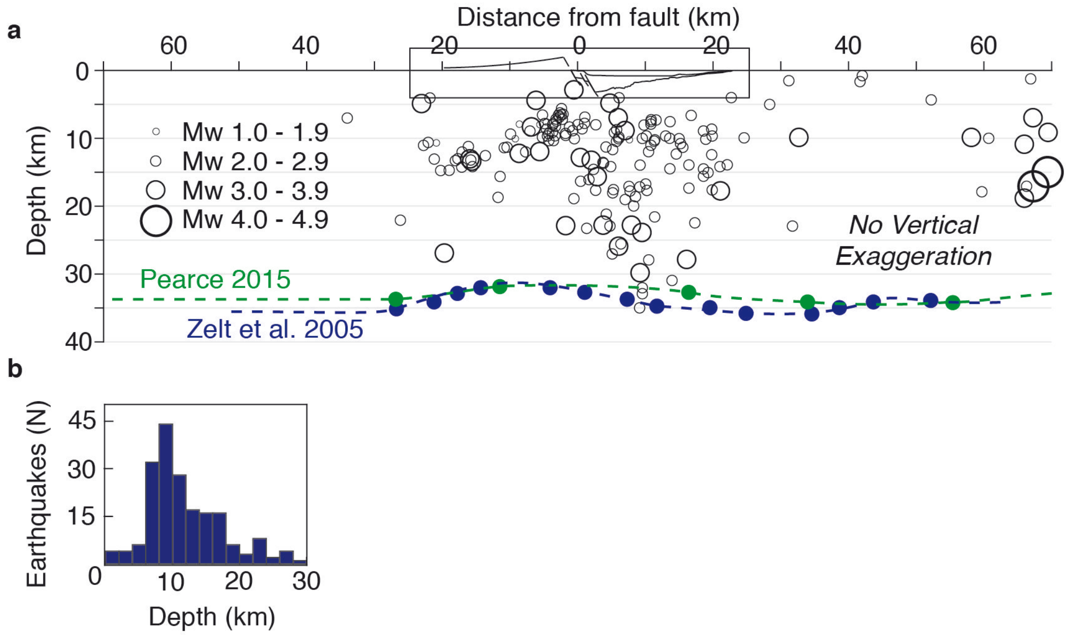

Supplementary Figure 6: Crustal scale cross-section. (a) Microseismicity from the University of Athens 1996-2008 earthquake catalogue measured within 2.5 km of profile A-A' in Fig. 1 and Moho depth estimates from Ps receiver functions[67] and tomographic inversion of PmP reflection times[68] **(b)** Histogram of (micro-)earthquake depths.

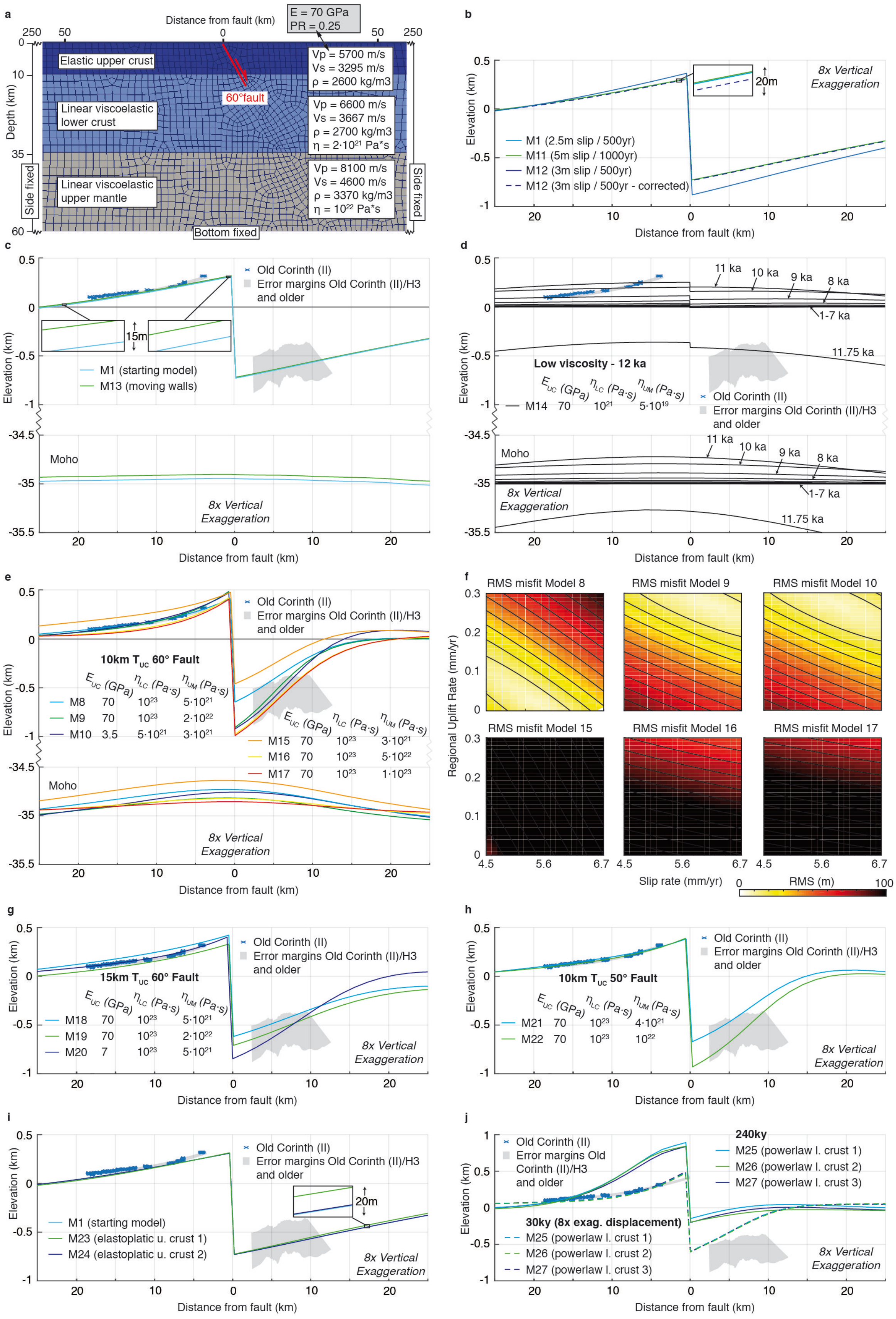
a
Distance from fault (km)
E = 70 GPa
PR = 0.25
250
50
0
50
250
Depth (km)
Elastic upper crust
Vp = 5700 m/s
Vs = 3295 m/s
ρ = 2600 kg/m3
60°fault
Linear viscoelastic lower crust
Vp = 6600 m/s
Vs = 3667 m/s
ρ = 2700 kg/m3
η = 2·10²¹ Pa*s
Linear viscoelastic upper mantle
Vp = 8100 m/s
Vs = 4600 m/s
ρ = 3370 kg/m3
η = 10²² Pa*s
Side fixed
Side fixed
Bottom fixed
b
Elevation (km)
8x Vertical Exaggeration
20m
M1 (2.5m slip / 500yr)
M11 (5m slip / 1000yr)
M12 (3m slip / 500yr)
M12 (3m slip / 500yr - corrected)
Distance from fault (km)
c
Old Corinth (II)
Error margins Old Corinth (II)/H3 and older
15m
M1 (starting model)
M13 (moving walls)
Moho
8x Vertical Exaggeration
Elevation (km)
Distance from fault (km)
d
11 ka
10 ka
9 ka
8 ka
1-7 ka
11.75 ka
Low viscosity - 12 ka
E_UC (GPa) η_LC (Pa·s) η_UM (Pa·s)
M14 70 10²¹ 5·10¹⁹
Old Corinth (II)
Error margins Old Corinth (II)/H3 and older
Moho
8x Vertical Exaggeration
Distance from fault (km)
e
Old Corinth (II)
Error margins Old Corinth (II)/H3 and older
10km T_uc 60° Fault
E_UC (GPa) η_LC (Pa·s) η_UM (Pa·s)
M8 70 10²³ 5·10²¹
M9 70 10²³ 2·10²²
M10 3.5 5·10²¹ 3·10²¹
M15 70 10²³ 3·10²¹
M16 70 10²³ 5·10²²
M17 70 10²³ 1·10²³
Moho
8x Vertical Exaggeration
Elevation (km)
Distance from fault (km)
f
RMS misfit Model 8
RMS misfit Model 9
RMS misfit Model 10
RMS misfit Model 15
RMS misfit Model 16
RMS misfit Model 17
Regional Uplift Rate (mm/yr)
Slip rate (mm/yr)
RMS (m)
g
Old Corinth (II)
Error margins Old Corinth (II)/H3 and older
15km T_uc 60° Fault
E_UC (GPa) η_LC (Pa·s) η_UM (Pa·s)
M18 70 10²³ 5·10²¹
M19 70 10²³ 2·10²²
M20 7 10²³ 5·10²¹
8x Vertical Exaggeration
Elevation (km)
Distance from fault (km)
h
Old Corinth (II)
Error margins Old Corinth (II)/H3 and older
10km T_uc 50° Fault
E_UC (GPa) η_LC (Pa·s) η_UM (Pa·s)
M21 70 10²³ 4·10²¹
M22 70 10²³ 10²²
8x Vertical Exaggeration
Elevation (km)
Distance from fault (km)
i
Old Corinth (II)
Error margins Old Corinth (II)/H3 and older
20m
M1 (starting model)
M23 (elastoplatic u. crust 1)
M24 (elastoplatic u. crust 2)
8x Vertical Exaggeration
Elevation (km)
Distance from fault (km)
j
Old Corinth (II)
Error margins Old Corinth (II)/H3 and older
240ky
M25 (powerlaw l. crust 1)
M26 (powerlaw l. crust 2)
M27 (powerlaw l. crust 3)
30ky (8x exag. displacement)
M25 (powerlaw l. crust 1)
M26 (powerlaw l. crust 2)
M27 (powerlaw l. crust 3)
8x Vertical Exaggeration
Elevation (km)
Distance from fault (km)

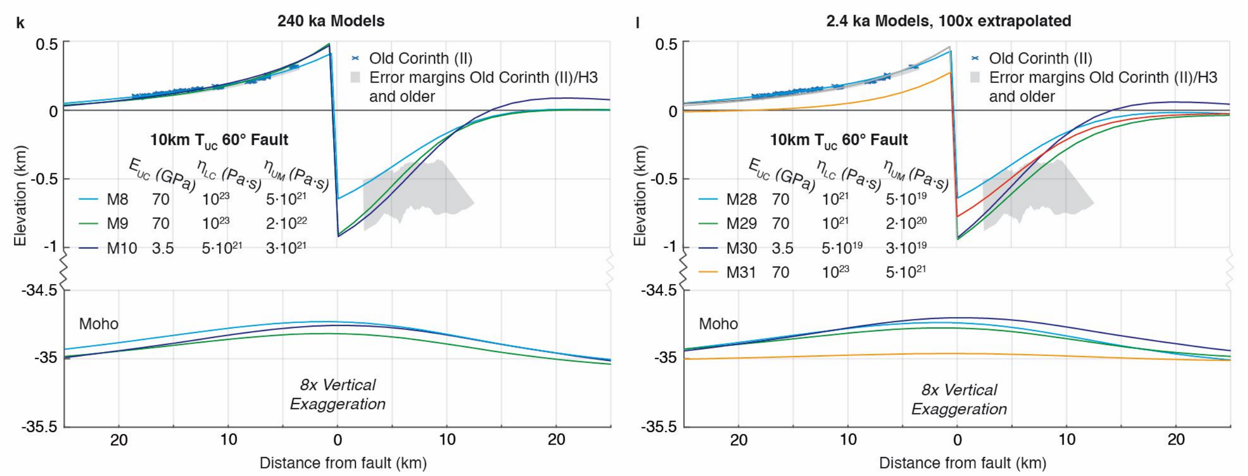

Supplementary Figure 7 (including previous page): Additional fault modelling results. (a) Subset of finite element grid showing Model 1 set-up. E = Young's Modulus, PR = Poisson's Ratio, Vp = P-wave velocity, Vs = S-wave velocity, ρ = density, η = viscosity **(b)** Tests on the influence of different earthquake recurrence time (M11) and slip rate (M12), the latter both uncorrected and with total displacement corrected by a factor 0.83 **(c)** Model with the same parameters as Model 1, but with 0.125 mm·yr^{-1} laterally moving sidewalls, and bottomwall moving upwards with 0.03 mm·yr^{-1} to isostatically compensate for lithospheric thinning **(d)** Example of model with two orders of magnitude lower viscosities with respect to M8, with timesteps plotted for every 1000 years until the model stops running after ~12 ka **(e)** Models 8-10 from Fig. 5b for comparison with g-l, including models with too low (M15) and too high (M16, M17) upper mantle viscosities for comparison **(f)** Root-mean-squared misfits of models 8-10 and 15-17 under the assumption of different fault slip rates and regional uplift rates. **(g)** Models with a 15km thick upper crust and 60° fault **(h)** Models with a 10 km thick upper crust and 50° fault **(i)** Models with an elastoplastic upper crust compared to Model 1 **(j)** Models with an elastoplastic upper crust and non-linear (powerlaw) viscoelastic lower crust compared to Model 1. **(k)** Repetition of Fig. 5c for comparison **(l)** Model results for viscosity values two orders of magnitude lower than M8-10 (M28-30) and the same as M8 (M31), but on a 2.4 ka timescale, plotted with a 100 times extrapolation of the surface deformation pattern.

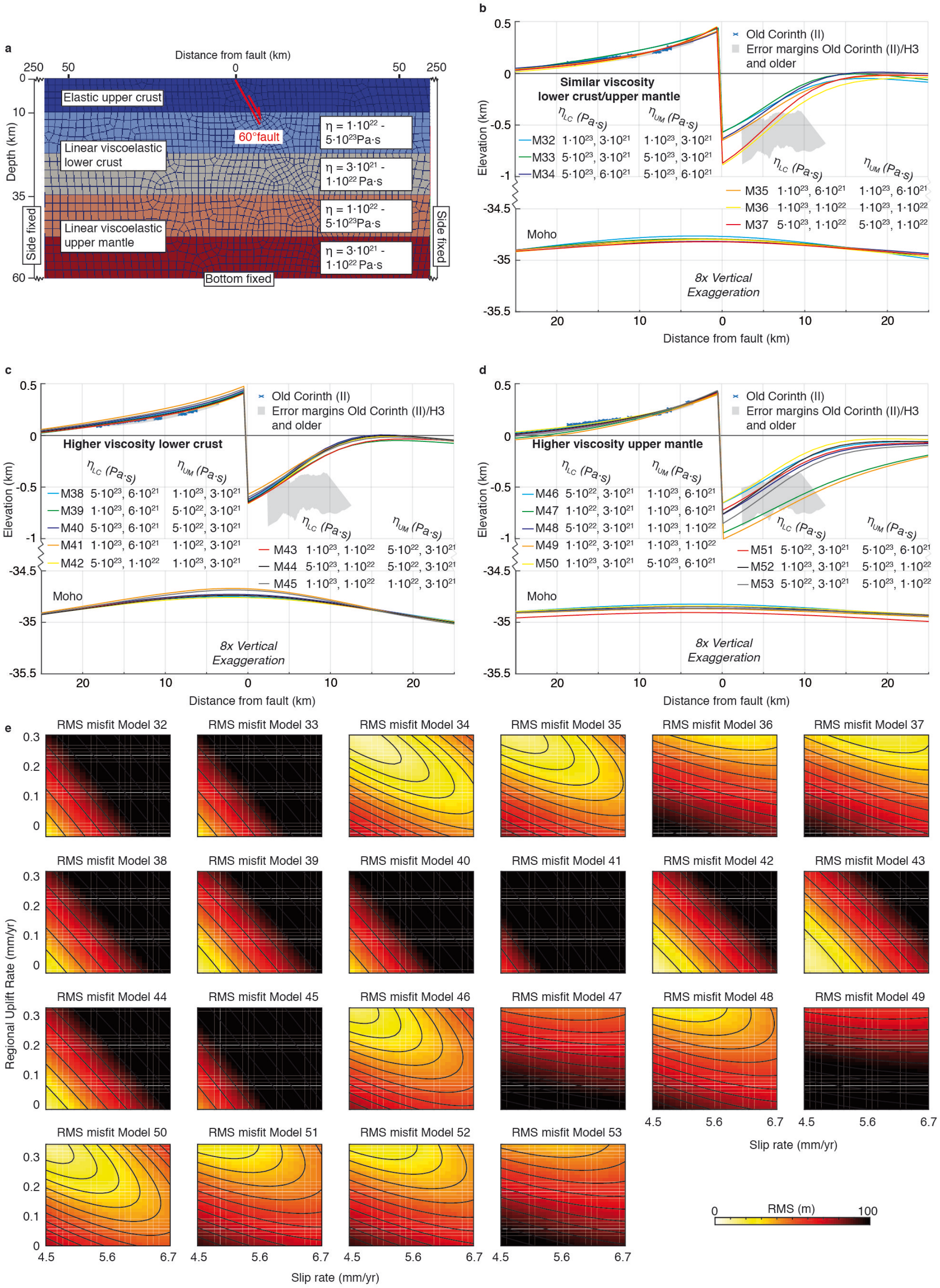

Supplementary Figure 8: Additional fault modelling for 5-layer models (a) Subset of finite element grid showing 5-layer model set-up. Fault angle, Upper crustal Young's Modulus and upper crustal thickness are the same as in M1 **(b-d)** Results for models with the same (**b**), higher (**c**) and lower (**d**) lower crustal viscosity with respect to the upper mantle. All models are plotted with the regional uplift rates

and slip rates that correspond to their minimum root-mean-squared misfit (see **e** and Supplementary Table 2) **(e)** Root-mean-squared misfits of models 32-53 under the assumption of different fault slip rates and regional uplift rates.

Table S1. Input parameters for numerical fault models

Elastic upper crust – linear viscoelastic lower crust – linear viscoelastic upper mantle

Model Nr.	Young's Modulus U.Crust (GPa)	Upper Crustal Thickness (km)	Fault Angle (°)	L. Crustal Viscosity (Pa·s)	U. Mantle Viscosity (Pa·s)
1	70	10	60	$2{\cdot}10^{21}$	10^{22}
2	7	10	60	$2{\cdot}10^{21}$	10^{22}
3	70	15	60	$2{\cdot}10^{21}$	10^{22}
4	70	10	40	$2{\cdot}10^{21}$	10^{22}
5	70	10	60	10^{22}	10^{22}
6	70	10	60	$2{\cdot}10^{21}$	10^{23}
7*	70	10	60	$2{\cdot}10^{21}$	10^{22}
8	70	10	60	10^{23}	$5{\cdot}10^{21}$
9	70	10	60	10^{23}	$2{\cdot}10^{22}$
10	3.5	10	60	$5{\cdot}10^{21}$	$3{\cdot}10^{21}$
11**	70	10	60	$2{\cdot}10^{21}$	10^{22}
12***	70	10	60	$2{\cdot}10^{21}$	10^{22}
13****	70	10	60	$2{\cdot}10^{21}$	10^{22}
14	70	10	60	10^{21}	$5{\cdot}10^{19}$
15	70	10	60	10^{23}	$3{\cdot}10^{21}$
16	70	10	60	10^{23}	$5{\cdot}10^{22}$
17	70	10	60	10^{23}	10^{23}
18	70	15	60	10^{23}	$5{\cdot}10^{21}$
19	70	15	60	10^{23}	$2{\cdot}10^{22}$
20	7	15	60	10^{23}	$5{\cdot}10^{21}$
21	70	10	50	10^{23}	$4{\cdot}10^{21}$
22	70	10	50	10^{23}	$8{\cdot}10^{21}$

* Same parameters as model 1, but with a fault crosscutting the whole crust at a 60° angle
** Same parameters as model 1, but with 5m slip earthquakes every 1000 years instead of 2.5m slip earthquakes every 500 years
*** Same parameters as model 1, but with 3m slip earthquakes every 500 years instead of 2.5m slip earthquakes every 500 years
**** Same parameters as model 1, but with walls laterally moving 0.125 mm/yr at both sides of the model, and upwards with 0.03 mm/yr at the bottom of the model

Elastoplastic upper crust – linear viscoelastic lower crust – linear viscoelastic upper mantle

Model Nr.	Cohesion (MPa)	Internal friction angle (°)	Dilatation angle (°)
23*	10	20	20
24*	50	30	30

* All other parameters same as model 1

Elastoplastic upper crust – non-linear (powerlaw) viscoelastic lower crust – linear viscoelastic upper mantle

Model Nr.	Temperature lower crust (°C)	Powerlaw stress exponent	Activation energy Q ($kJ{\cdot}mol^{-1}$)	Pre-exponential term A ($MPa^{-n}{\cdot}s^{-1}$)	U. Mantle Viscosity (Pa·s)
25*	300-720	4.0	223	$1.1{\cdot}10^{-4}$	10^{22}
26*	300-650	4.0	223	$1.1{\cdot}10^{-4}$	10^{22}
27*	300-720	4.0	223	$1.1{\cdot}10^{-4}$	10^{23}

* All other parameters same as model 18

Table S2. Viscosities and misfits for numerical fault models

3-Layer models with 10 km crustal thickness, 60° fault angle, and 70 GPa Upper Crustal Young's Modulus

Model Nr.	L. Crustal Viscosity 10 – 35 km (Pa·s)	U. Mantle Viscosity 35 – 60 km (Pa·s)	Plotted slip rate ($mm \cdot yr^{-1}$)	Plotted regional uplift rate ($mm \cdot yr^{-1}$)	RMS misfit (m)
15	$1 \cdot 10^{23}$	$3 \cdot 10^{21}$	4.5	0.0	85.7
8	$1 \cdot 10^{23}$	$5 \cdot 10^{21}$	5.1	0.01	7.4
9	$1 \cdot 10^{23}$	$2 \cdot 10^{22}$	5.5	0.27	7.1
16	$1 \cdot 10^{23}$	$5 \cdot 10^{22}$	6.7	0.3	52.2
17	$1 \cdot 10^{23}$	$1 \cdot 10^{23}$	6.7	0.3	63.9

5-Layer models with 10 km crustal thickness, 60° fault angle, and 70 GPa Upper Crustal Young's Modulus

Model Nr.	L. Crustal Viscosity 10 – 22.5 km (Pa·s)	L. Crustal Viscosity 22.5 – 35 km(Pa·s)	U. Mantle Viscosity 35 – 47.5 km (Pa·s)	U. Mantle Viscosity 47.5 – 60 km (Pa·s)	Plotted slip rate ($mm \cdot yr^{-1}$)	Plotted regional uplift rate ($mm \cdot yr^{-1}$)	RMS misfit (m)
32	$1 \cdot 10^{23}$	$3 \cdot 10^{21}$	$1 \cdot 10^{23}$	$3 \cdot 10^{21}$	4.5	0.0	27.4
33	$5 \cdot 10^{23}$	$3 \cdot 10^{21}$	$5 \cdot 10^{23}$	$3 \cdot 10^{21}$	4.5	0.0	28.7
34	$5 \cdot 10^{23}$	$6 \cdot 10^{21}$	$5 \cdot 10^{23}$	$6 \cdot 10^{21}$	4.6	0.3	8.4
35	$1 \cdot 10^{23}$	$6 \cdot 10^{21}$	$1 \cdot 10^{23}$	$6 \cdot 10^{21}$	4.7	0.3	11.4
36	$1 \cdot 10^{23}$	$1 \cdot 10^{22}$	$1 \cdot 10^{23}$	$1 \cdot 10^{22}$	5.9	0.3	25.8
37	$5 \cdot 10^{23}$	$1 \cdot 10^{22}$	$5 \cdot 10^{23}$	$1 \cdot 10^{22}$	5.8	0.3	16.7
38	$5 \cdot 10^{23}$	$6 \cdot 10^{21}$	$1 \cdot 10^{23}$	$3 \cdot 10^{21}$	4.5	0.0	19.3
39	$1 \cdot 10^{23}$	$6 \cdot 10^{21}$	$5 \cdot 10^{22}$	$3 \cdot 10^{21}$	4.5	0.0	14.9
40	$5 \cdot 10^{23}$	$6 \cdot 10^{21}$	$5 \cdot 10^{22}$	$3 \cdot 10^{21}$	4.5	0.0	29.2
41	$1 \cdot 10^{23}$	$6 \cdot 10^{21}$	$1 \cdot 10^{22}$	$3 \cdot 10^{21}$	4.5	0.0	60.1
42	$5 \cdot 10^{23}$	$1 \cdot 10^{22}$	$1 \cdot 10^{23}$	$3 \cdot 10^{21}$	4.5	0.0	9.5
43	$1 \cdot 10^{23}$	$1 \cdot 10^{22}$	$5 \cdot 10^{22}$	$3 \cdot 10^{21}$	4.5	0.04	10.8
44	$5 \cdot 10^{23}$	$1 \cdot 10^{22}$	$5 \cdot 10^{22}$	$3 \cdot 10^{21}$	4.5	0.0	14.0
45	$1 \cdot 10^{23}$	$1 \cdot 10^{22}$	$1 \cdot 10^{22}$	$3 \cdot 10^{21}$	4.5	0.0	40.9
46	$5 \cdot 10^{22}$	$3 \cdot 10^{21}$	$1 \cdot 10^{23}$	$6 \cdot 10^{21}$	4.5	0.3	14.8
47	$1 \cdot 10^{22}$	$3 \cdot 10^{21}$	$1 \cdot 10^{23}$	$6 \cdot 10^{21}$	5.6	0.3	43.8
48	$5 \cdot 10^{22}$	$3 \cdot 10^{21}$	$1 \cdot 10^{23}$	$1 \cdot 10^{22}$	5.0	0.3	24.7
49	$1 \cdot 10^{22}$	$3 \cdot 10^{21}$	$1 \cdot 10^{23}$	$1 \cdot 10^{22}$	5.8	0.3	58.0
50	$1 \cdot 10^{23}$	$3 \cdot 10^{21}$	$5 \cdot 10^{23}$	$6 \cdot 10^{21}$	4.5	0.3	11.4
51	$5 \cdot 10^{22}$	$3 \cdot 10^{21}$	$5 \cdot 10^{23}$	$6 \cdot 10^{21}$	4.8	0.3	21.4
52	$1 \cdot 10^{23}$	$3 \cdot 10^{21}$	$5 \cdot 10^{23}$	$1 \cdot 10^{22}$	5.0	0.3	19.1
53	$5 \cdot 10^{22}$	$3 \cdot 10^{21}$	$5 \cdot 10^{23}$	$1 \cdot 10^{22}$	5.4	0.3	33.6
